# Supplementary figures and images for: Spatial Congruence Analysis (SCAN): A method for detecting biogeographical patterns based on species range congruences
Source: PLoS One. 2021 May 20;16(5):e0245818. doi: 10.1371/journal.pone.0245818 (PMC8136640; doi:10.1371/journal.pone.0245818)

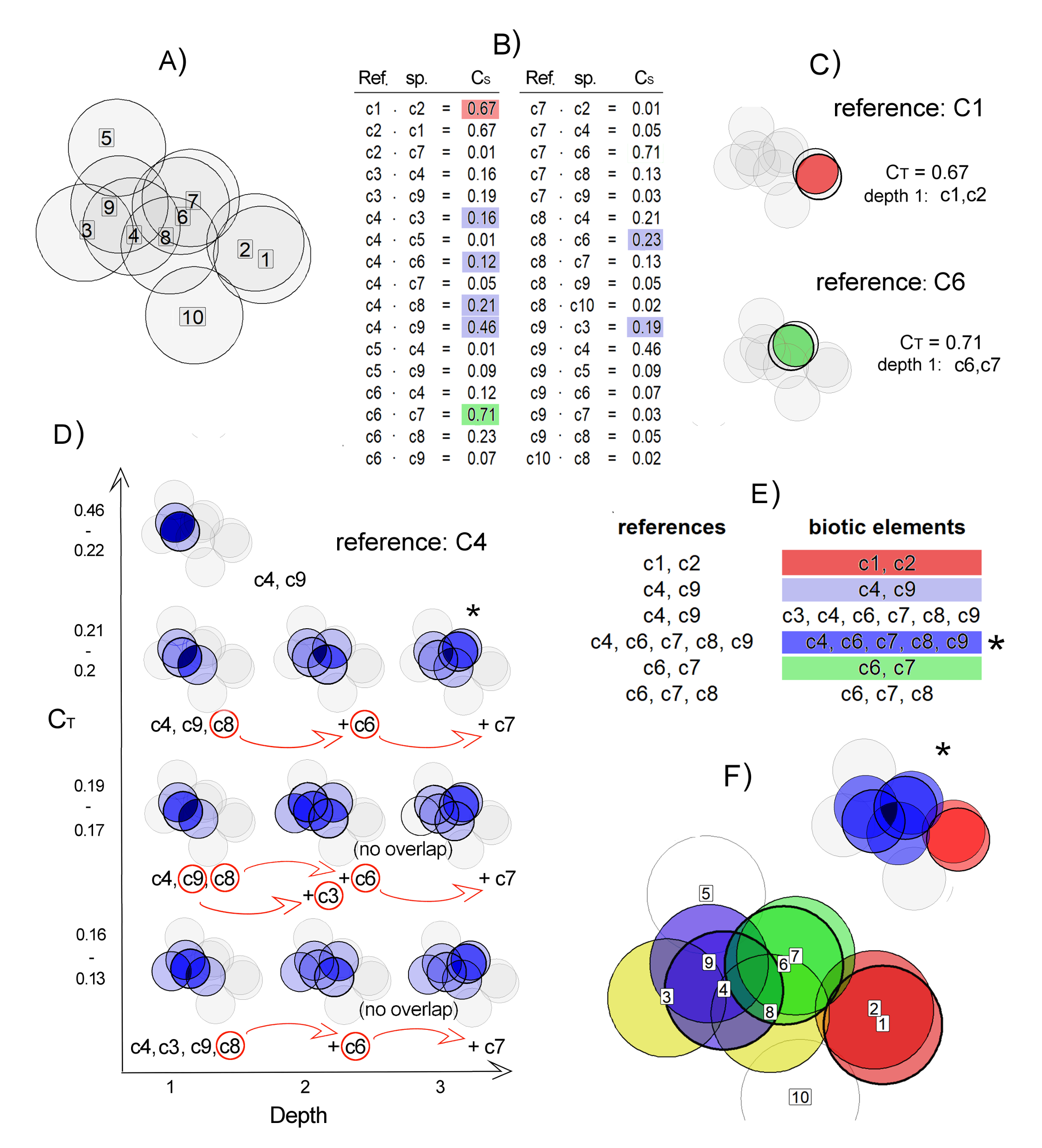

Supplement: S1 Fig — (A) Spatial representation of a hypothetical regional community of ten simulated species (c1-c10). (B) Congruence similarities between all overlapping species pairs (CS ≥ 0.01) are used for all direct and indirect comparisons in the following steps. (C) At relatively high congruence thresholds (CT), as references species, c1 and c6 each gives rise to only one partial chorotype (closed list) composed of two directly-related species (CT = 0.67 and 0.71, respectively). (D) Depending on CT, c4 may have numerous indirect relationships. At CT between 0.46 and 0.22, it is only (directly) connected to c9. As of 0.21, c4 is also directly connected (depth 1) to c8 which links (indicated with arrow) to c6 (depth 2), which in turn links to c7 (depth 3). This partial chorotype (indicated with an asterisk) will be further examined in subsequent examples. At 0.19, an additional link, between c9 and c3, appears at depth 2. However, with this suite of ranges, there is no longer any area of overlap among all ranges (shown in black when it occurs); the lack of range overlap would cause the analysis to drop this CT round. (E) All informative reference species and their respective partial chorotypes recovered (colored as in preceding figures). Ranges c5 and c10 were not included in any patterns, and c3 was included in some groups, but did not give rise to partial chorotypes. The pattern [c4+c9+c8+c6+c7] can be derived from any of its constituent species (dark blue; asterisk). (F) Possible classification schemes show the trade-off between congruence and comprehensiveness. A highly congruent scheme has three independent two-species patterns (blue, green, and red patterns), and c3 and c8 are out (yellow). A less congruent scheme (asterisk) has a very comprehensive pattern (blue) based on less congruent indirect links, with a common area for all ranges. It encompasses many nested sub-patterns (shown as distinct colors in the previous classification). (TIF) [file pone.0245818.s001.tif]

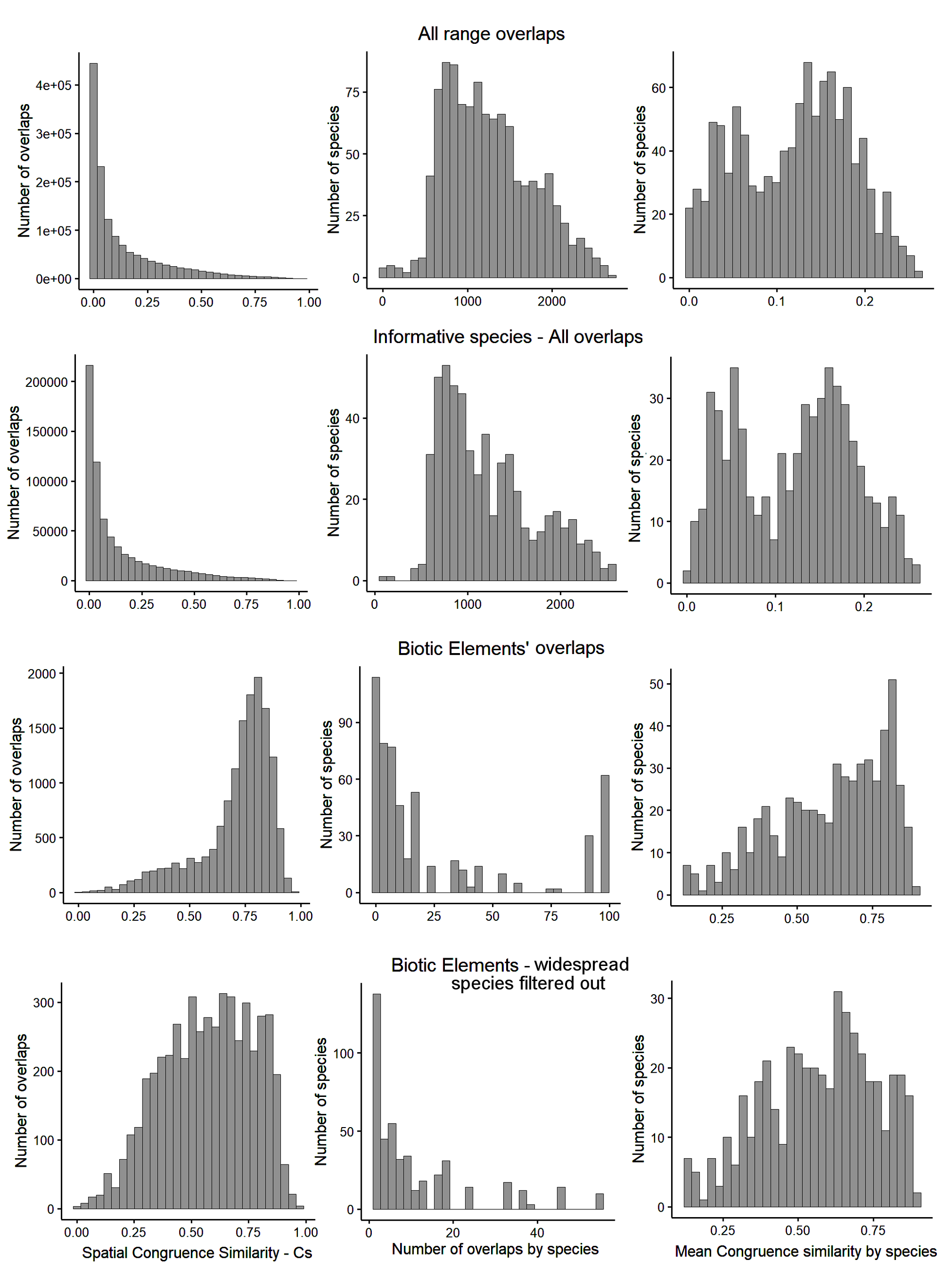

Supplement: S2 Fig — When all range overlaps of the avifauna are considered (first row), most have low spatial congruence similarity values (first column; mean±sd = 0.13±0.18, n = 1.37M overlaps), a high number of overlaps by species (second column; 1252±520, n = 1095 species), and low CS means (third column; 0.12±0.06, n = 1095 species). The same pattern is repeated for all overlaps of informative species (second row; n = 519 species). When only overlaps composing chorotypes are considered (third row) the pattern is inverted. CS values are high (0.71±0.17; n = 14597 overlaps of 558 species) for a small number of overlaps (26±34, n = 558) with higher mean CS (0.61±0.19, n = 558). When widespread and non-Amazonian chorotypes are filtered out (forth row) the pattern is modified with slightly lower means (0.57±0.2, n = 5088), overlaps (11.1±12.8, n = 457), and CS (0.57±0.18,n = 457). (TIF) [file pone.0245818.s002.tif]

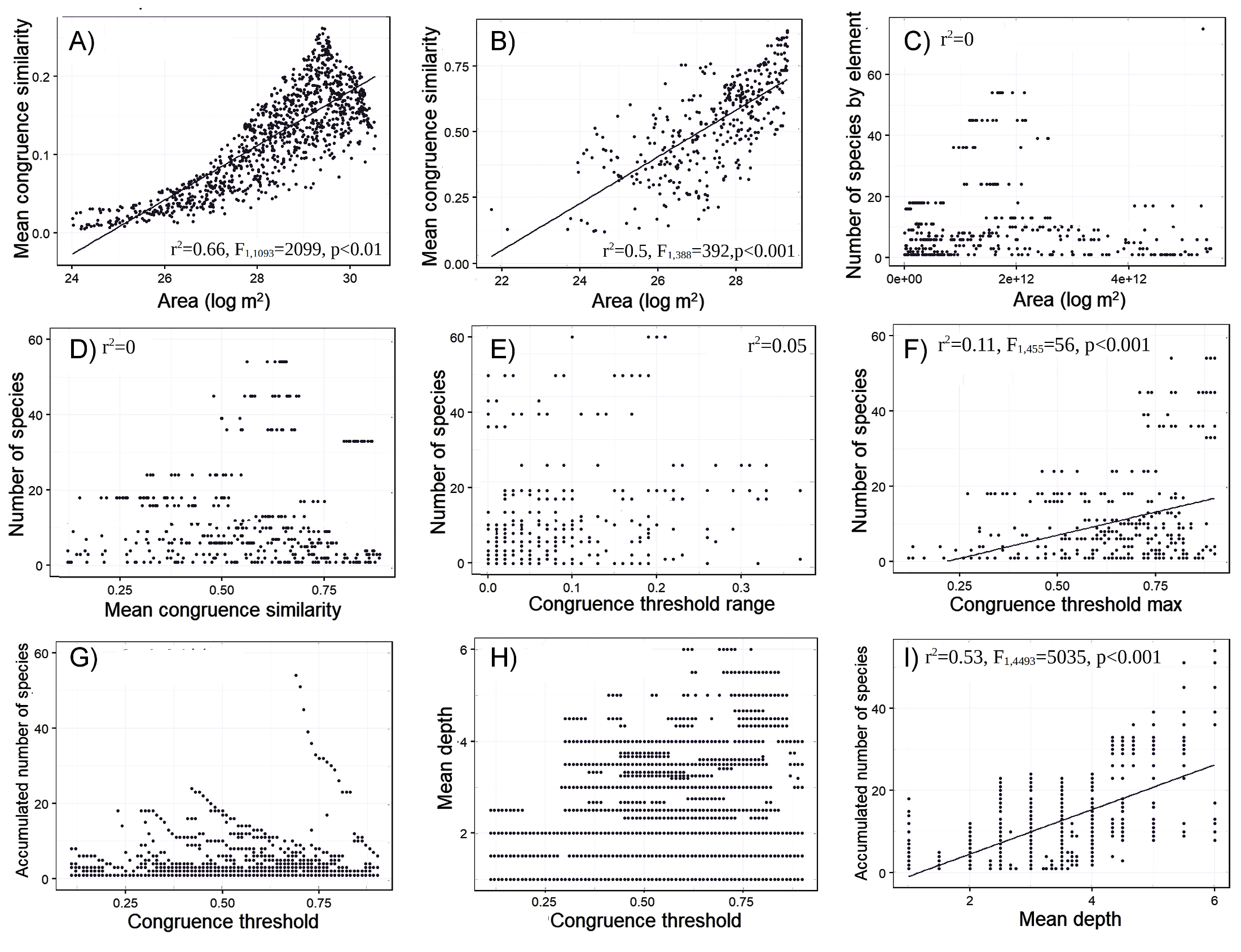

Supplement: S3 Fig — From (B) to (J) two disproportionately large patterns (one gathering 99 transcontinental, and another 92 widespread taxa all across Amazonia) were filtered out. In general, ordinary linear regression analyses (OLS) showed that taxa with larger ranges have higher CS means (A, r2 = 0.66, F1,1093 = 2099, p<0.01), even for partial chorotypes (B, r2 = 0.5, F1,388 = 392,p<0.001). However, patterns derived from larger ranges are not richer (C, r2 = 0). The richness of a chorotype is not related to the mean CS (D, r2 = 0), and only negligible effects from spam (E, r2 = 0.05, F1,455 = 26, p<0.001) or maximum threshold values (CT) were detected (F, r2 = 0.11, F1,455 = 56, p<0.001). For all chorotypes, CT has a negligible positive effect over the number of species and mean depth (not shown, r2 = 0). However, if reference species are modeled as random effects in linear mixed models, lower CT allow more species (G, Fixed effect: accumulated number of species Ct, r2 = 0.84, F1,4473 = 2780, p<0.001; Random effect: reference species, Var = 157.9±12.1, Res = 15.8, Var ratio = 9.98, 90% explained), at lower mean depths (H, Fixed effect: mean depth, r2 = 0.94, F1,4473 = 472, p<0.001; Random effect: reference species, Var = 1.89±0.13, Res = 0.11, Var ratio = 17, 94.5% explained). Depth is positively related with species richness (I; OLS, r2 = 0.53, F1,4493 = 5035, p<0.001). Some data distributions may violate premises of OLS regressions, such as heteroscedasticity: we opted to kept these distributions as they are (i.e., non-transformed) to allow a better visualization of these distributions in their natural shapes. In this context, statistical significance and regression coefficients may be seen only from an exploratory perspective. (TIF) [file pone.0245818.s003.tif]
