# Supplementary material for: Spatial Congruence Analysis (SCAN): A method for detecting biogeographical patterns based on species range congruences
Source: PLoS One. 2021 May 20;16(5):e0245818. doi: 10.1371/journal.pone.0245818 (PMC8136640; doi:10.1371/journal.pone.0245818)
Supplement: S1 Table — Among the 30 ‘species’ analyzed as references, the algorithm recognized 27 as biogeographically informative. Although smaller, the maximum depth setting of 3 (Max-depth) allowed the recognition of highly congruent patterns, as shown by the maximum and minimum congruence thresholds. These partial chorotypes are enough to classify the gradient into 5 distinct non-overlapping zones (Fig 3A), which gain more species and expand at lower congruences (Fig 3B). More relaxed depth settings allow larger groups at intermediate congruences with larger chains of indirect connections. All 23 unique partial chorotypes recovered are nested to one of the patterns grouping all species of the South or North (11 and 12 patterns, respectively; Fig 3D). Partial chorotypes depicted in Fig 3 are referenced in the last column. (RTF) [file pone.0245818.s004.rtf]

S1 Table. Biogeographic elements in the simulated hypothetical gradient. Among the 30 ‘especies’f analyzed as references, the algorithm recognized 27 as biogeographically informative. Although smaller, the maximum depth setting of 3 (Max-depth) allowed the recognition of highly congruent patterns, as shown by the maximum and minimum congruence thresholds. These elements are enough to classify the gradient into 5 distinct non-overlapping zones (Fig 3A), which gain more species and expand at lower congruences (Fig 3B). More relaxed depth settings allow larger groups at intermediate congruences with larger chains of indirect connections. All 23 unique biogeographic elements recovered are nested to one of the patterns grouping all species of the South or North (11 and 12 patterns, respectively; Fig 3D). Patterns depicted in Fig 3 are referenced in the last column.
