# Supplementary material for: Spatial Congruence Analysis (SCAN): A method for detecting biogeographical patterns based on species range congruences
Source: PLoS One. 2021 May 20;16(5):e0245818. doi: 10.1371/journal.pone.0245818 (PMC8136640; doi:10.1371/journal.pone.0245818)
Supplement: S2 Table — All possible partial chorotypes for each reference species at each distinct max-depth configuration (3, 5, 7, and 10). Number of species, spatial congruence similarity, and max. and min. values of threshold and depth for each chorotype are presented. (RTF) [file pone.0245818.s005.rtf]

S2 Table. Simulated hypothetical gradient of ranges by maximum depth settings. Biogeographic elements for each reference species at each distinct max-depth configuration (3, 5, 7, and 10). Number of species, spatial congruence similarity, and max. and min. values of threshold and depth for each biogeographic element are presented.
max depth set	Ref. species	n spp	Cs mean	Ct max	Ct min	depth max	depth min	species of the larger biotic element		
3	S8	4	0.87	0.90	0.86	2	2	S6, S7, S9, S10		
3	S9	3	0.90	0.90	0.88	2	1	S7, S8, S10		
3	S10	2	0.88	0.90	0.89	2	2	S8, S9		
3	S13	4	0.92	0.94	0.85	2	1	S11, S12, S14, S15		
3	S14	4	0.92	0.94	0.85	2	1	S11, S12, S13, S15		
3	S15	2	0.93	0.94	0.94	2	2	S13, S14		
3	N16	2	0.93	0.94	0.94	2	2	N17, N18		
3	N17	4	0.92	0.94	0.85	2	1	N16, N18, N19, N20		
3	N18	4	0.92	0.94	0.85	2	1	N16, N17, N19, N20		
3	N21	2	0.88	0.90	0.89	2	2	N22, N23		
3	N22	3	0.90	0.90	0.88	2	1	N21, N23, N24		
3	N23	4	0.87	0.90	0.86	2	2	N21, N22, N24, N25		
3	N28	2	0.73	0.80	0.51	2	1	N29, N30		
3	N29	2	0.78	0.80	0.51	1	1	N28, N30		
3	N30	2	0.69	0.75	0.51	2	1	N28, N29		
5	S2	14	0.29	0.50	0.12	4	2	S1, S3, S4, S5, S6, S7, S8, S9, S10, S11, S12, S13, S14, S15		
5	S6	5	0.79	0.87	0.85	4	4	S5, S7, S8, S9, S10		
5	S7	5	0.84	0.88	0.85	3	3	S5, S6, S8, S9, S10		
5	S8	5	0.86	0.90	0.85	3	2	S5, S6, S7, S9, S10		
5	S9	5	0.87	0.90	0.85	4	1	S5, S6, S7, S8, S10		
5	S10	4	0.85	0.90	0.86	4	2	S6, S7, S8, S9		
5	S11	4	0.81	0.86	0.85	3	3	S12, S13, S14, S15		
5	S12	4	0.90	0.93	0.85	3	2	S11, S13, S14, S15		
5	S13	4	0.92	0.94	0.85	2	1	S11, S12, S14, S15		
5	S14	4	0.92	0.94	0.85	2	1	S11, S12, S13, S15		
5	S15	4	0.90	0.94	0.85	3	2	S11, S12, S13, S14		
5	N16	4	0.90	0.94	0.85	3	2	N17, N18, N19, N20		
5	N17	4	0.92	0.94	0.85	2	1	N16, N18, N19, N20		
5	N18	4	0.92	0.94	0.85	2	1	N16, N17, N19, N20		
5	N19	4	0.90	0.93	0.85	3	2	N16, N17, N18, N20		
5	N20	4	0.81	0.86	0.85	3	3	N16, N17, N18, N19		
5	N21	4	0.85	0.90	0.86	4	2	N22, N23, N24, N25		
5	N22	5	0.87	0.90	0.85	4	1	N21, N23, N24, N25, N26		
5	N23	5	0.86	0.90	0.85	3	2	N21, N22, N24, N25, N26		
5	N24	5	0.84	0.88	0.85	3	3	N21, N22, N23, N25, N26		
5	N25	5	0.79	0.87	0.85	4	4	N21, N22, N23, N24, N26		
5	N28	14	0.53	0.80	0.12	3	1	N16, N17, N18, N19, N20, N21, N22, N23, N24, N25, N26, N27, N29, N30		
5	N29	14	0.54	0.80	0.12	4	1	N16, N17, N18, N19, N20, N21, N22, N23, N24, N25, N26, N27, N28, N30		
5	N30	14	0.45	0.75	0.12	4	1	N16, N17, N18, N19, N20, N21, N22, N23, N24, N25, N26, N27, N28, N29		
max depth set	Ref. species	n spp	Cs mean	Ct max	Ct min	depth max	depth min	species of the larger biotic element		
7	S1	14	0.18	0.50	0.12	5	2	S2, S3, S4, S5, S6, S7, S8, S9, S10, S11, S12, S13, S14, S15		
7	S2	14	0.29	0.50	0.12	4	2	S1, S3, S4, S5, S6, S7, S8, S9, S10, S11, S12, S13, S14, S15		
7	S5	5	0.74	0.85	0.85	5	5	S6, S7, S8, S9, S10		
7	S6	5	0.79	0.87	0.85	4	4	S5, S7, S8, S9, S10		
7	S7	5	0.84	0.88	0.85	3	3	S5, S6, S8, S9, S10		
7	S8	14	0.68	0.90	0.12	6	2	S1, S2, S3, S4, S5, S6, S7, S9, S10, S11, S12, S13, S14, S15		
7	S9	14	0.68	0.90	0.12	5	1	S1, S2, S3, S4, S5, S6, S7, S8, S10, S11, S12, S13, S14, S15		
7	S10	14	0.68	0.90	0.12	6	2	S1, S2, S3, S4, S5, S6, S7, S8, S9, S11, S12, S13, S14, S15		
7	S11	10	0.79	0.86	0.84	6	3	S5, S6, S7, S8, S9, S10, S12, S13, S14, S15		
7	S12	4	0.90	0.93	0.85	3	2	S11, S13, S14, S15		
7	S13	4	0.92	0.94	0.85	2	1	S11, S12, S14, S15		
7	S14	4	0.92	0.94	0.85	2	1	S11, S12, S13, S15		
7	S15	4	0.90	0.94	0.85	3	2	S11, S12, S13, S14		
7	N16	4	0.90	0.94	0.85	3	2	N17, N18, N19, N20		
7	N17	4	0.92	0.94	0.85	2	1	N16, N18, N19, N20		
7	N18	4	0.92	0.94	0.85	2	1	N16, N17, N19, N20		
7	N19	4	0.90	0.93	0.85	3	2	N16, N17, N18, N20		
7	N20	10	0.79	0.86	0.84	6	3	N16, N17, N18, N19, N21, N22, N23, N24, N25, N26		
7	N21	14	0.68	0.90	0.12	6	1	N16, N17, N18, N19, N20, N22, N23, N24, N25, N26, N27, N28, N29, N30		
7	N22	14	0.69	0.90	0.12	5	1	N16, N17, N18, N19, N20, N21, N23, N24, N25, N26, N27, N28, N29, N30		
7	N23	14	0.68	0.90	0.12	6	1	N16, N17, N18, N19, N20, N21, N22, N24, N25, N26, N27, N28, N29, N30		
7	N24	5	0.84	0.88	0.85	3	3	N21, N22, N23, N25, N26		
7	N25	5	0.79	0.87	0.85	4	4	N21, N22, N23, N24, N26		
7	N26	5	0.74	0.85	0.85	5	5	N21, N22, N23, N24, N25		
7	N28	14	0.53	0.80	0.12	3	1	N16, N17, N18, N19, N20, N21, N22, N23, N24, N25, N26, N27, N29, N30		
7	N29	14	0.54	0.80	0.12	4	1	N16, N17, N18, N19, N20, N21, N22, N23, N24, N25, N26, N27, N28, N30		
7	N30	14	0.45	0.75	0.12	4	1	N16, N17, N18, N19, N20, N21, N22, N23, N24, N25, N26, N27, N28, N29		
10	S1	14	0.18	0.50	0.12	5	2	S2, S3, S4, S5, S6, S7, S8, S9, S10, S11, S12, S13, S14, S15		
10	S2	14	0.29	0.50	0.12	4	2	S1, S3, S4, S5, S6, S7, S8, S9, S10, S11, S12, S13, S14, S15		
10	S3	14	0.47	0.80	0.12	9	1	S1, S2, S4, S5, S6, S7, S8, S9, S10, S11, S12, S13, S14, S15		
10	S4	14	0.54	0.83	0.12	9	1	S1, S2, S3, S5, S6, S7, S8, S9, S10, S11, S12, S13, S14, S15		
10	S5	14	0.59	0.85	0.12	9	1	S1, S2, S3, S4, S6, S7, S8, S9, S10, S11, S12, S13, S14, S15		
10	S6	14	0.63	0.87	0.12	8	1	S1, S2, S3, S4, S5, S7, S8, S9, S10, S11, S12, S13, S14, S15		
10	S7	14	0.66	0.88	0.12	7	1	S1, S2, S3, S4, S5, S6, S8, S9, S10, S11, S12, S13, S14, S15		
10	S8	14	0.68	0.90	0.12	6	2	S1, S2, S3, S4, S5, S6, S7, S9, S10, S11, S12, S13, S14, S15		
10	S9	14	0.68	0.90	0.12	5	1	S1, S2, S3, S4, S5, S6, S7, S8, S10, S11, S12, S13, S14, S15		
10	S10	14	0.68	0.90	0.12	6	2	S1, S2, S3, S4, S5, S6, S7, S8, S9, S11, S12, S13, S14, S15		
10	S11	14	0.66	0.86	0.12	7	2	S1, S2, S3, S4, S5, S6, S7, S8, S9, S10, S12, S13, S14, S15		
10	S12	14	0.67	0.93	0.12	8	2	S1, S2, S3, S4, S5, S6, S7, S8, S9, S10, S11, S13, S14, S15		
10	S13	14	0.67	0.94	0.12	9	1	S1, S2, S3, S4, S5, S6, S7, S8, S9, S10, S11, S12, S14, S15		
10	S14	14	0.65	0.94	0.12	9	1	S1, S2, S3, S4, S5, S6, S7, S8, S9, S10, S11, S12, S13, S15		
10	S15	14	0.63	0.94	0.12	9	2	S1, S2, S3, S4, S5, S6, S7, S8, S9, S10, S11, S12, S13, S14		
10	N16	14	0.64	0.94	0.12	9	2	N17, N18, N19, N20, N21, N22, N23, N24, N25, N26, N27, N28, N29, N30		
10	N17	14	0.67	0.94	0.12	9	1	N16, N18, N19, N20, N21, N22, N23, N24, N25, N26, N27, N28, N29, N30		
10	N18	14	0.68	0.94	0.12	9	1	N16, N17, N19, N20, N21, N22, N23, N24, N25, N26, N27, N28, N29, N30		
10	N19	14	0.69	0.93	0.12	8	2	N16, N17, N18, N20, N21, N22, N23, N24, N25, N26, N27, N28, N29, N30		
10	N20	14	0.67	0.86	0.12	7	2	N16, N17, N18, N19, N21, N22, N23, N24, N25, N26, N27, N28, N29, N30		
10	N21	14	0.68	0.90	0.12	6	1	N16, N17, N18, N19, N20, N22, N23, N24, N25, N26, N27, N28, N29, N30		
10	N22	14	0.69	0.90	0.12	5	1	N16, N17, N18, N19, N20, N21, N23, N24, N25, N26, N27, N28, N29, N30		
10	N23	14	0.68	0.90	0.12	6	1	N16, N17, N18, N19, N20, N21, N22, N24, N25, N26, N27, N28, N29, N30		
10	N24	14	0.66	0.88	0.12	7	1	N16, N17, N18, N19, N20, N21, N22, N23, N25, N26, N27, N28, N29, N30		
10	N25	14	0.63	0.87	0.12	8	1	N16, N17, N18, N19, N20, N21, N22, N23, N24, N26, N27, N28, N29, N30		
10	N26	14	0.58	0.85	0.12	9	1	N16, N17, N18, N19, N20, N21, N22, N23, N24, N25, N27, N28, N29, N30		
10	N27	14	0.52	0.83	0.12	9	1	N16, N17, N18, N19, N20, N21, N22, N23, N24, N25, N26, N28, N29, N30		
10	N28	14	0.53	0.80	0.12	3	1	N16, N17, N18, N19, N20, N21, N22, N23, N24, N25, N26, N27, N29, N30		
10	N29	14	0.54	0.80	0.12	4	1	N16, N17, N18, N19, N20, N21, N22, N23, N24, N25, N26, N27, N28, N30		
10	N30	14	0.45	0.75	0.12	4	1	N16, N17, N18, N19, N20, N21, N22, N23, N24, N25, N26, N27, N28, N29		
