# Supplementary material for: Spatial Congruence Analysis (SCAN): A method for detecting biogeographical patterns based on species range congruences
Source: PLoS One. 2021 May 20;16(5):e0245818. doi: 10.1371/journal.pone.0245818 (PMC8136640; doi:10.1371/journal.pone.0245818)
Supplement: S3 Table — The congruence algorithm groups species with direct (depth 1) and indirect (depth > 1) relationships to the reference at each congruence threshold (CT). CS index shows the lower CT which allows a direct relationship between the grouped species and the reference. CT max and min show the range of CT values in which the group species compose the partial chorotype. For example, the Icterus nigrogularis chorotype at CT = 0.73 (CT max) (follow CT max and depth values at Fig 4A) has Tyrannus dominicensis directly related (CS = 0.73); this species links the reference to Hydropsalis cayennensis (depth = 2), which has a direct congruence to the reference of only CS = 0.71 (lower than the current threshold). At CT max = 0.42 the last species joins the group (Inezia caudata). Taxonomy and distribution follow BirdLife and HBW [46]. (RTF) [file pone.0245818.s006.rtf]

S3 Table. Biogeographic complexes derived from selected illustrative South American bird species. The congruence algorithm groups species with direct (depth 1) and indirect (depth > 1) relationships to the reference at each congruence threshold (CT). CS index shows the lower CT which allows a direct relationship between the grouped species and the reference. CT max and min show the range of CT values in which the Group species compose the biotic complex. For example, the Icterus nigrogularis complex at CT=0.73 (CT max) (follow CT max and depth values at Fig 4A) has Tyrannus dominicensis directly related (CS=0.73); this species links the reference to Hydropsalis cayennensis (depth=2), which has a direct congruence to the reference of only CS=0.71 (lower than the current threshold). At CT max=0.42 the last species joins the group (Inezia caudata). Taxonomy and distribution follow BirdLife and HBW (2016).


	
