# Supplementary material for: Spatial Congruence Analysis (SCAN): A method for detecting biogeographical patterns based on species range congruences
Source: PLoS One. 2021 May 20;16(5):e0245818. doi: 10.1371/journal.pone.0245818 (PMC8136640; doi:10.1371/journal.pone.0245818)
Supplement: S4 Table — Species sharing biogeographical properties converge to synonym chorotypes, in which parameters, and spatial and taxonomic composition are mostly alike. Nested chorotypes are taxonomic subsets of a larger pattern. The patterns shown at Figs 4 and 5 (references species in bold) can be generated by other species grouped at their respective chorotypes. Usually the higher CS mean to all other members gives the name to the chorotype. The only exception is Heliodoxa xanthogonys, which here, for convenience, gives its name to the ‘Tepuis1’ pattern. This is the only presented chorotype with nested subsets (1.1, 1.1.1). For patterns at the Amazonian margins, not all species composing the groups (S1 Table) matched the criteria of inclusion, and were not analyzed as references (e.g, some Heliodoxa spp. chorotypes). (RTF) [file pone.0245818.s007.rtf]

S4 Table. Bird's synonym and nested biogeographic complexes. Species sharing biogeographical properties converge to synonym complexes, in which parameters, and spatial and taxonomic composition are mostly alike. Nested complexes are taxonomic subsets of larger pattern. The patterns shown at Figs 4 and 5 (references species in bold) can be generated by other species grouped at their respective biogeographic complexes. Usually the higher CS mean to all other members gives the name to the pattern. The only exception is Heliodoxa xanthogonys, which here, for convenience, gives its name to the 'Tepuis1' pattern. This is the only presented complex with nested subsets (1.1, 1.1.1). For patterns at the Amazonian margins, not all species composing the groups (S1 Table) matched the criteria of inclusion, and were not analyzed as references (e.g, some Heliodoxa spp. complexes).
