# Supplementary material for: Spatial Congruence Analysis (SCAN): A method for detecting biogeographical patterns based on species range congruences
Source: PLoS One. 2021 May 20;16(5):e0245818. doi: 10.1371/journal.pone.0245818 (PMC8136640; doi:10.1371/journal.pone.0245818)
Supplement: S1 Script — This script is meant to be maintained and updated at https://github.com/cassianogatto/congruence_source. To use this version, save the following script (code lines) as a ‘source_congruence_1.1.R’ document in the main directory of your analyzes, and ‘source’ it using the following code: “source (“C:/my_directory/…/source_congruence_1.1.R’)”; to edit the script load it on an editor, such as RStudio https://rstudio.com/products/rstudio/). (RTF) [file pone.0245818.s008.rtf]

Spatial Congruence Analysis (SCAN): A method for detecting biogeographical patterns based on species’ range congruences
Cassiano AFR Gatto & Mario Cohn-Haft

S1 Script. Source code to the congruence framework functions. This script is meant to be maintaned and updated at https://github.com/cassianogatto/congruence_source. To use this version, 
save this text as a ‘source_congruence_1.1.R’ document in the main directory of your analyzes and ‘source’ it. 
source(“C:/my_directory/.../source_congruence_1.1.R’
(to edit the script load it on an editor, such as RStudio https://rstudio.com/products/rstudio/)
library(sf)
library(tidyverse)
library(ggplot2)
library(ggmap)
# library(googleway) # (only for specific graphs)
# some basic settings
examples = FALSE
`%notin%` = Negate(`%in%`)

#### Main functions ####

# FUNCTION SP_LEVELS_BASED_ON_SIMILARITIES
# 'similarity levels: apply congruence threshold over a set of n fixed levels – results are CLOSED (if species are repeated in successive levels – Biotic Elements), or OPEN systems'
# 'from version 9.5 added map_species to inter_patt_levels'
# 'requires functions: inter_patt_level and inter_patt'
# 'Column names sim_df are species1, species2, dist_sim (for Csim)'
sp_levels_sim = function (sp_focus, sim_df = sim_df_ae,
                          map_species = mapAE_SA,
                          max_sim = 1, min_sim = 0.75, n_levels = 5,
                          save_intersect = TRUE,
                          new_window = FALSE, row_col = c(2,5), background_map = sa, abbrev = F,
                          intersection_plot = TRUE,...) {
# sp_focus is a species or a vector of species or a data frame with SCINAME column
    print('sp_levels_sim_V2.2') # intersection_plot = FALSE option  -> fast and clean closed/open system check
     
    # checks, data frames, create lists, etc
    if(is.vector(sp_focus)){  sp_df = data_frame('SCINAME'= sp_focus)  
    } else { if(is.data.frame(sp_focus)) { sp_df = sp_focus %>% select(SCINAME) } else {print('wrong type of sp input'); break  
    }}
    list_spp = list()
    unique_list_spp = data.frame()
    #check column Csim to dist_sim
    if ('Csim' %in% names(sim_df) & !('dist_sim' %in% names(sim_df)) ){sim_df = sim_df %>% select(species1,species2, dist_sim = Csim, everything())}
    
    list_spp[['info']] = paste(' sp_sim_levels V1 - congruence in a max number of levels (interactions)', '/n',
                                  'pattern derived from', nrow(sp_df), 'species:', sp_focus, '/n',
                                  'with max', max_sim, 'and min', min_sim, 'and ', n_levels, 'levels of analysis,','/n',
                                  ifelse(intersection_plot,'with plot and calculus of intersections','with NO plot and calculus of intersections'))
    level_down = list_spp[['pattern']] # I didn't create the object before but it keeps just with the reference? hmmm how da hell is it workin'? Prob NOT! :/
    status = 'OPEN'
    if(intersection_plot){   if(new_window){ x11(); par(mar=c(2,5,2,2), mfrow = row_col)   }    }
    
    # LOOP LEVELS - N_LEVELS - remember the 'trick' : each time sp_df reload, it cames with
    #  the last list loaded...
    #  so its a feedback in itself and the way the list grows in each cycle of the loop
    for (n in 1:n_levels){
        sp_df = sim_df %>% select(species1,species2,dist_sim) %>% filter(species1 %in% sp_df[,'SCINAME']) %>% 
            filter(as.numeric(dist_sim) >= as.numeric(min_sim) & as.numeric(dist_sim) <= max_sim) %>% 
            mutate('dist_sim' = round(as.numeric(dist_sim),3)) %>% arrange(desc(dist_sim)) %>%
            select(species1,species2) %>% gather %>% select(-key, 'SCINAME' = value) %>% unique %>% as.data.frame
        level = paste ('level', n, sep='')
        
        list_spp[[level]] = sp_df      # if someone wants individual level lists turn it on - it makes the final object a lit bit too confuse but makes things easier to plot
        
        print(paste(min_sim, ' level ', n))
        print(sp_df)
        # comparison between species in this with previous level - is the same set of species?
        if (all(unique(sp_df[,'SCINAME']) %in% unique(level_down[,'SCINAME']))) {
            status = 'CLOSED'
            list_spp[['status']] = paste ('CLOSED - after ', n-1,'levels',sep=' ')
            print(paste ('CLOSED - after ',n-1,'levels, with', length(unique(sp_df[,'SCINAME'])),'species',sep=' ')); break }
        
        list_spp[['species_level']] = rbind(list_spp[['species_level']], data.frame('SCINAME' = sp_df, 'level' = n)) %>% arrange(SCINAME,level)
        level_down = sp_df # backup copy for subsequent comparison
        title_fcn = paste ( ifelse(abbrev,abbrev(sp_focus[1]), sp_focus),' ',round(min_sim,2), '\nlevel ', n, ', with ',
                           length(unique(sp_df[,'SCINAME'])),' species',sep='')

# intersection & plots
        if (intersection_plot){     # if inter_patt = T  APPLY FUNCTION INTER_PATT inter_patt and PLOT maps and save the intersection calculated at this level
            
            inter_patt_level = inter_patt(sp_df = sp_df, map_species =  map_species, title = title_fcn, new_window = F, 
                                          background_map = background_map, plot_background = TRUE)
            
            # inter_patt is a list with 'intersect' & 'union'

            if (n > 1) { intersect_level = st_intersection(inter_patt_level[['intersect']] , intersect_level)  # make it cumulative across levels but I dont think is necessary - I already fixed the problem in inter_patt - pls check last version
            }  else { intersect_level = inter_patt_level[['intersect']] }
            
            assign(paste('inter_level', n, sep=''), intersect_level)    # modified 13Feb2019
            
            if (n > 1) { union_level = st_union(inter_patt_level[['union']], union_level)        # make it cumulative
            }  else  { union_level = inter_patt_level[['union']] }                    ## added 03/jan/19  # modified 12 fev 2019
            
            # calculus of intersection/union ratio per level # added 9/jan/2019
            list_spp[['inter_union_ratio']][[paste('level', n)]] = st_area(intersect_level)/ st_area(union_level)
            # calculus of the intersect area loss after new level species
            if (n > 1) {
                inter_last_level = get(paste('inter_level', n-1, sep=''))
                list_spp[['inter_loss']][[paste('level', n)]] = st_area(intersect_level)/ st_area(inter_last_level) }
            # break no intersection
            if (is.null(intersect_level)) { list_spp[['status']] = paste ('NO INTERSECTION on the level', n)
            print(paste ('NO INTERSECTION in the ', n,'level', sep=' '))
            break }
        } else {  print('No intersection evaluation and no plots. If needed pls use intersection_plot = TRUE')
            list_spp[['inter_loss']][[paste('level', n)]] = paste('no intersection calculus in this mode')        }
        _________    
        
        if (n == n_levels){ list_spp[['status']] = paste ('OPEN - after ',n,'levels with',length(unique(sp_df[,'SCINAME'])),'species',sep=' ')
        print(paste ('OPEN - after ', n,'levels with', length(unique(sp_df[,'SCINAME'])),'species', sep=' ')) }
        
        unique_list_spp =  list_spp[['species_level']] #rbind(unique_list_spp,sp_df) %>% unique
        
    } # for loop ends here
    # create a line to convert many lists to one data frame here
    # do.call("rbind", lapply(your_list, data.frame, stringsAsFactors=FALSE))
    if (intersection_plot){     if(save_intersect) { list_spp[['intersection']] = intersect_level; list_spp[['union']] = union_level }   }
    
    if(!intersection_plot){ list_spp = cbind(unique_list_spp, data.frame('min_sim'= rep(min_sim, nrow(unique_list_spp)),
                                                                         'n_lev'= rep(ifelse(status == 'CLOSED', n-1, n_levels),nrow(unique_list_spp)),
                                                                         'status'= rep(status, nrow(unique_list_spp)))) 
    list_spp = unique(list_spp) %>% arrange(desc(min_sim),level,SCINAME)
    }
    return(list_spp)
}
# if(examples){
#     test = sp_levels_sim(sp_focus = 'Capito auratus', sim_df = sim_df, max_sim = 1, min_sim = 0.6, n_levels = 4,
#                          save_intersect = TRUE, new_window = TRUE, row_col = c(1,4), background_map = sa)
# }

# 'find the congruence threshold between a closed and an open system for all species in a list'
# 'a data frame is generated with species in each level of congruence and status (open, closed), etc'
# 'use a data frame with species in a "SCINAME" column. tab_min excludes status==OPEN data...'
# 'requires functions: sp_levels_sim (requires inter_patt)'
# 'source_clean_V9.5 removed "areas" and "tab_min" options - these transformations can be made outside via summary, left_join with area_df, etc.'

threshold_levels = function (spp_df, min_sim_init = .9, min_sim_step = .01, n_levels = 6,
                             sim_df = sim_df_lowlands, map_species = mapAE_SA,...){
    
    print('this is threshold_levels V1.3')# no areas or tab_min #print('this is threshold_levels V1.2')# V1.1 with minimal data frame (summarised) as result option #V 1.2 improvement in final df
    threshold_results = data.frame()
    threshold_tab_min = data.frame()
    min_sim_init_backup = min_sim_init
    if (!is.data.frame(spp_df)){print('spp_df needs to be a dataframe with species in SCINAME col'); break }
    if (all(names(spp_df) != 'SCINAME')){
        if (any(names(spp_df) == "species2")) { spp_df = spp_df %>% select('SCINAME' = species2)
        } else { spp_df = spp_df %>% select('SCINAME' = species1) }
    }
    
    for(i in 1: nrow(spp_df)){
        sp_focus = as.character(spp_df$SCINAME[i]);    sp_focus;      print(paste('progress ', i/nrow(spp_df)*100,'%'))
        #setup
        min_sim_init = min_sim_init_backup
        status = 'CLOSED'
        # loop status closed
        while(status == 'CLOSED'){
            if(min_sim_init < .1) {print('min_sim < .1'); break} # don't know why sometimes things go to negative loops minsim
            print(min_sim_init)
            
#___call_function_sp_levels_sim
            list_spp_congruence_min = sp_levels_sim (sp_focus = sp_focus, sim_df = sim_df,
                                        max_sim = 1, min_sim = min_sim_init, n_levels = n_levels,
                                        intersection_plot = FALSE, map_species = map_species)
            #filter only the lower level for each congruence value
            # list_spp_congruence_min = list_spp_congruence_min %>% group_by(SCINAME) %>% filter(level == min(level)) %>% as.data.frame

            # while there is no spp > min_sim
            if (nrow(list_spp_congruence_min) == 0) { 
                min_sim_init = min_sim_init - min_sim_step; next  }  #criteria to decrease min_sim_init just works to intersection_plot = FALSE format
            
            #if OPEN break to next species without saving
            if (unique(list_spp_congruence_min$status)=='OPEN'){ break }
            if(nrow(list_spp_congruence_min) > 0){ list_spp_congruence_min = list_spp_congruence_min %>% filter(SCINAME != sp_focus) }
            
            # with species list > 0
            threshold_results_partial = cbind('sp_focus' = as.character(rep(sp_focus, nrow(list_spp_congruence_min))), list_spp_congruence_min)
            threshold_results_partial = threshold_results_partial %>% mutate_if(is.factor, as.character)
                        
            threshold_results = rbind(threshold_results, threshold_results_partial)
            status = threshold_results_partial[nrow(threshold_results_partial), 'status'] 
            
            print('#'); print('##');  print('###')
            print(paste(sp_focus, min_sim_init, status)) # changed to status
            # status and min_sim_init set up for next round inner to while
            
            min_sim_init = min_sim_init - min_sim_step
            
            if(min_sim_init < .1) {print('min_sim < .1'); break}
            
            # > names(threshold_results)
            # [1] "sp_focus" "SCINAME"  "level"    "min_sim"  "n_lev"    "status" 
            
        } 
    }  # for loop ends here
    
    threshold_results = threshold_results %>% filter(status == 'CLOSED') %>%  filter(sp_focus != SCINAME) %>% 
        select(species1 = sp_focus, species2 = SCINAME, Cthres = min_sim, depth = level, status)
    
    return(threshold_results)
}

# if(examples){
#     l.serena = threshold_levels(spp_df = data.frame('species1'=c('Lepidothrix serena','Capito niger')), min_sim_step=.05 ,sim_df = sim_df_lowlands,tab_min=TRUE)
#     
#     thres_test = threshold_levels(spp_df = r.melano.73[1:3,], min_sim_init = .85, min_sim_step = .02, n_levels = 6)
# }

# 'coherence function'
# 'description:' 
# {'This function takes a species as focus and plots species across a range
#   of values of coherence; uses a similarity data frame, similarity max and min,
#   and allows the choice of method to set congruence thresholds intervals (decimal (step=.1), user defined interval levels, or fixed n levels).'
#   'position = c("internal_to","overlap","contains") runs the internal function (sp_int_ext) and chooses
#   only the selected categories - gives the status on the data frame printed and saved on object'
#   'results in a list with species by level and if save_shapefiles saves final intersection and union'  
#     'if  accum_intersection it will plot intersection and union accummulated across levels - otherwise 
#     plot_spp_sim_maps will plot level inter and union'
#   
#   'dependencies: functions: plot_spp_sim_maps, bb_max_spp'}
coherence_to_sp = function(sp_focus, sim_df = sim_df_ae, max_sim = 1, min_sim = 0.2, map_species = mapAE_SA, 
                           method = 'decimal', #method = 'n_levels', n_levels = 5 ,  # method = 'fixed', fixed_levels = c(.99,.8,.7,.6), # default is the same as decimal
                           accum_intersection = TRUE,
                           position = c('overlap','contains', 'internal_to', NA), #  position = FALSE #(faster but with no internal external status)
                           n_levels = 5, # method = 'n_levels', # n_levels = trunc((max_sim-min_sim)*10), # option 
                           background_map = sa, row_col = c(2,4), 
                           save_shapefiles = TRUE, new_window = TRUE,
                                   name_abbrev = TRUE, min_to_overlap = .95, iu_title = TRUE, ...  ){
        print('coherence_to_sp V2.5') # small adjusts in accum_intersection here and in plot_spp_sim_maps - vide comments above
             
        tab_spp = sim_df %>% filter(species1 == sp_focus) %>% filter (species2!= sp_focus) %>% 
                select(SCINAME = species2, dist_sim) %>% filter(dist_sim >= min_sim & dist_sim <= max_sim) %>%
                mutate_if(is.factor,as.character) %>% arrange(desc(dist_sim)) %>% as.data.frame
        if(nrow(tab_spp)==0) stop('unable to build a table with the current parameters')
            
        if (position[1] != FALSE){ # df internal external overlap (can control overlay criterion through 'min_to_overlap' if used at main function arguments)
            tab_spp1 = sp_int_ext (sp_focus = sp_focus, tab_spp = tab_spp, map = map_species,
                                   min_to_overlap = ifelse(exists('min_to_overlap'),min_to_overlap,.95));
            tab_spp =  tab_spp1 %>% filter(status_sp_focus %in% position) %>% select(SCINAME, dist_sim, status_sp_focus, everything()) %>% as.data.frame() # !! everything
        }
        sp_map = map_species %>% filter(SCINAME == sp_focus)
        intersection = sp_map
        union = sp_map
        results = list()
        IU_ratio_accum_level = 1
        if(!exists('method')) { method = 'decimal' }
        results[['details']] = paste(sp_focus, min_sim,'-', max_sim,' method:', method, ifelse(method == 'n_levels', paste('n_levels=', n_levels),
                ifelse(method == 'fixed', paste('levels=', fixed_levels), ifelse((method == 'decimal' | method == 'dec'), paste('.1 intervals'),''))))
        results[['species_list']] = data_frame()
        # print(results[['species_list']])
        bb_max_tab_spp = bb_max_spp(tab_spp, map_base = map_species)
        # print(bb_max_tab_spp)
        if(new_window){x11();par(mfrow = row_col, mar = c(3,3,3,2))}
        # define intervals of of sim by method and its parameters
        if (method == 'decimal' | method == 'dec') {  group_lvl = trunc(tab_spp$dist_sim * 10)  }
        if (method == 'n_levels' | method == 'n_lev') {    #group_lvl = split(tab_spp$SCINAME, cut( seq_along (tab_spp$SCINAME),n_levels, labels = FALSE)) # here just to consult options to split
            group_lvl = ntile(tab_spp$dist_sim, n_levels) } # same as above but simpler with ntile n_levels = 4 is 'quantile'
        if (method == 'fixed'){    group_lvl = cut( tab_spp$dist_sim,fixed_levels,labels=FALSE)  }
        
        ## plots with the function plot_spp_sim_maps
        for(ii in (as.numeric(unique(group_lvl)))){ # TEST tab_spp_lvl = tab_spp[which(group_lvl==ii),];print(tab_spp_lvl)}
            tab_spp_lvl = tab_spp[which(group_lvl==ii),]
            lvl_max_sim = tab_spp_lvl$dist_sim %>% max %>% round(.,3)
            lvl_min_sim = tab_spp_lvl$dist_sim %>% min %>% round(.,3)
            sp_abbrev = ifelse(name_abbrev, paste(str_sub(sp_focus,1,1),'.', word(sp_focus,2,2), sep = ''), sp_focus)
            # title & plot
            title = paste(sp_abbrev,'\n S:',lvl_max_sim,'-',lvl_min_sim,' N=', nrow(tab_spp_lvl),sep='') 
            print(title)
            print(tab_spp_lvl)
            
            plot(background_map$geometry, xlim = bb_max_tab_spp[c(1,3)], ylim = bb_max_tab_spp[c(2,4)])#, col=rgb(0,.1,0,1))
                  
        ### Two colored title :)
            if(iu_title){
                if(exists('IU_ratio_accum_level')) {
                    IU_title = paste('last_IU_ratio = ',round(as.numeric(IU_ratio_accum_level),2), sep='')
                    if(as.numeric(IU_ratio_accum_level) < .5) { 
                        title(paste(title,' ',IU_title), col.main = 'dark red')
                    } else {
                        title(paste(title,' ',IU_title), col.main = 'dark blue') 
                        }}} else {title(title)}
           
            ## select level species and apply function plot_sim_spp_maps
            Pv_plot = plot_spp_sim_maps(tab_spp = tab_spp_lvl, background_plot = FALSE, map_species = map_species, 
                                        plot_level_inter_union = !accum_intersection)
            if(IU_ratio_accum_level != 0) {intersection = intersection %>% st_intersection (Pv_plot[['intersection']])}
            union = union %>% st_union(Pv_plot[['union']])
            if (length(st_area(intersection)) == 0 ){IU_ratio_accum_level = 0 } else {
                IU_ratio_accum_level = as.numeric(st_area(intersection))/ as.numeric(st_area(union)) %>% as.numeric }
            
            plot(sp_map %>% st_geometry, lwd=1, add=T, col = rgb(0,0,0,.2))
            
            #results[['species_list']][[paste('LEVEL_',ii,' S:', lvl_max_sim,'-', lvl_min_sim, sep='')]] = tab_spp_lvl
            data = cbind(tab_spp_lvl, data_frame ('max_sim_level' = rep(lvl_max_sim, nrow(tab_spp_lvl)),
                                                  'min_sim_level' = rep(lvl_min_sim, nrow(tab_spp_lvl)),
                                                  'IU_accum_level' = rep(as.numeric(IU_ratio_accum_level), nrow(tab_spp_lvl))))
            results[['species_list']] = rbind(results[['species_list']],data)
            # saving only level values, NOT accumulated ones... needs to apply something like 'lapply function(x) inter = inter st_inter(x)' etc...
            if(save_shapefiles){results[['shapefiles']][[paste('level',ii)]] = list('intersection' = Pv_plot[['intersection']],'union' = Pv_plot[['union']])  }
        
            # plot intersection and union accumulated
            if(accum_intersection){ 
                plot(st_geometry(union), add = T, col = rgb(0,1,1,.01), lwd = 2, lty = 1)
                if(length(st_area(intersection)) != 0) {plot(st_geometry(intersection), add = T, col = rgb(.5,.5,0,.7), lwd = 2)}
            }
          }
          if(save_shapefiles){results[['intersection']] = intersection; results[['union']] = union}
          if(position[1] != FALSE) {results[['species_list']] = results[['species_list']] %>% select(sp_focus, status_sp_focus, SCINAME, 
                                        dist_sim, IU_sp_sp, area_sp_sp_ratio, IU_accum_level, max_sim_level, min_sim_level, everything() )}
          results
}

# ' sp_int_ext V1 - internal external'
# 'to calculate internal or external status of sp_focus compared to  other polygons in a df$SCINAME:'
# 'description'
# {'overlap -> when the congruence is >=.95 (default) or any value min_to_overlap'
#   'contains -> when the species focus contains > .98 of sp2 range'
#   'internal_to -> sp1 is >=.98 inside of sp2 range (overlay = 0.98 of sp1 range)'
#   'NA -> none of the above criteria applies'
#   "to filter use (.) %>% filter(status_sp_focus == 'internal_to' | status_sp_focus == 'contains') %>% as.data.frame()"
#   'V.9.5 map to map_species'}
sp_int_ext = function(sp_focus, tab_spp, map_species = mapAE_SA, 
                      min_to_overlap = .9, min_to_internal_to = .95, min_to_contains = .95,  # <<< --- parameters to internal, contains, overlap and NA !!!
                      full_df = FALSE,...){
  
    print('sp_int_ext V1.2') # IU ratio sp by sp / ratio sp by sp areas
  if(is.vector(tab_spp)) { tab_spp = data_frame('SCINAME' = tab_spp) }
  sp_map = map_species %>% filter(SCINAME == sp_focus)
  area_sp_map = st_area(sp_map)
  df_results = data_frame()
  for (i in 1:length(tab_spp$SCINAME)){
    map_sp2 = map_species %>% filter(SCINAME == tab_spp$SCINAME[i])
    area_sp2 = st_area(map_sp2)
    inter_area = st_intersection(sp_map,map_sp2) %>% st_area
    ov_1 = inter_area/area_sp_map
    ov_2 = inter_area/area_sp2
    area_union = st_area(st_geometry(st_union(sp_map,map_sp2)))
    # status verification
    if (as.numeric(ov_1*ov_2) >= min_to_overlap) {status_sp1 = 'overlap'} else {
      if (as.numeric(ov_1) > as.numeric(ov_2)){
        if (as.numeric(ov_1) >= min_to_internal_to) {status_sp1 = 'internal_to'} else {status_sp1 = NA}} else {
          if (as.numeric(ov_2) > min_to_contains) {status_sp1 = 'contains'}  else {status_sp1 = NA}}
      }
    # write df
    if(full_df){df_results = rbind(df_results, data_frame('sp_focus' = sp_focus, 'SCINAME' = tab_spp$SCINAME[i],
                                                             'status_sp_focus'= status_sp1, 'overlay' = inter_area,
                                                             'dist_sim' = as.numeric(round(ov_1*ov_2,3)),
                                                            'IU_sp_sp' = as.numeric(round(inter_area/area_union,3)),
                                                            'area_sp_sp_ratio' = as.numeric(round(area_sp_map/area_sp2,3)),
                                                            'area_sp1' = area_sp_map, 'area_sp2' = area_sp2 ))
    } else {df_results = rbind(df_results, data_frame('sp_focus' = sp_focus,'status_sp_focus'= status_sp1,
                                                     'SCINAME' = tab_spp$SCINAME[i],'dist_sim' = as.numeric(round(ov_1*ov_2,4)),
                                                     'IU_sp_sp' = as.numeric(round(inter_area/area_union,3)),
                                                     'area_sp_sp_ratio' = as.numeric(round(area_sp_map/area_sp2,3)))) } 
  }
  df_results %>% as.data.frame
}

# plot_spp_focus 27NOV2018
# 'plot sp focus in range sim max-min, choose between internal_to, overlap and contains in position parameter'
# 'Saved objects =  df_spp species list used to print'
# 'color_scheme = 1,2 or 3 are color gradiens; 4 is all blue and other options are direct, e.g. color_scheme = rgb(.5,.3,.7,.2)'
# 'methods = "sp_focus" or "spp_df" in which the data frame must have SCINAME, dist_sim, status_sp_focus & area column names'
plot_spp_focus = function ( method = 'sp_focus', # option method = 'spp_df'
                            sp_focus,  spp_df = spp_df, max_sim = 1, min_sim = .1, similarity_df = sim_df,
                            area_df = area_species,  position = FALSE, # plots everything and # position = c('contains', 'overlap') selects internal spp
                            map_species = mapAE_SA, backgroud_map = sa, new_window = FALSE, new_plot = TRUE, color_scheme = 1,...)  { # color_scheme = rgb(0,0,1,.005)
    # area_species = sim_df_lowlands %>% select(SCINAME = species1,area = area_sp1) %>% unique (can use to calculate areas)
  # print('plot_spp_focus_V1.1')#color_scheme
  print('plot_spp_focus_V1.2')#color_scheme == 4 (all blue), method = spp_df, etc 27/NOV/2018
  print(paste('method =',method))
  if (!exists("area_species")){area_df = area_species_SA}
  if (method != 'sp_focus' & method != 'spp_df'){ print('method must be specified: "sp_focus" or "spp_df"');break }
  if (method == 'sp_focus'){
    sp_df = sim_sp_df(sp_focus = sp_focus, max_sim = max_sim ,min_sim = min_sim)
    
    # int_ext parameters from plot_spp_focus
    sp_df = sp_int_ext(sp_focus = sp_focus, tab_spp = sp_df) # sp_int_ext to verify a df to position internal, external or overlay
    
    print(sp_focus)
    print(paste('N-spp: ',length(sp_df$SCINAME),sep=''))
    sp_df %>% group_by(status_sp_focus) %>% summarise(n=n()) %>% print
    #sort by area
    if(!is.character(area_df[,'SCINAME'])){area_df = area_df %>% mutate_if(is.factor,as.character)}
    if(!is.numeric(area_df[,'area'])){area_df[,'area'] = area_df %>% select(area) %>% mutate_if(is.character,as.double)}
    sp_df = sp_df %>% left_join(area_df, by=c('SCINAME'='SCINAME')) %>% arrange(desc(area))
    # filter species overlap or contains
    if(position[1] != FALSE) {sp_df = sp_df %>% filter(status_sp_focus %in% position) %>% as.data.frame() }
  }
  if (method == 'spp_df'){
    if (!exists('sp_focus')) {sp_focus = 'species focus'}
    if (all(c('SCINAME','dist_sim','status_sp_focus','area') %in% names(spp_df))){ sp_df = spp_df;
    sp_df = sp_df %>% arrange(desc(area))
    } else { print('columns spp_df must be "SCINAME","dist_sim","status_sp_focus" & "area"')}
  }
  #sort by area
  
  # plot 
  if(new_window) {x11()}
  bb_max = bb_max_spp(sp_df, map_base = map_species)
  if(new_plot) {
    title = paste( paste(str_sub(sp_focus,1,1),'.', word(sp_focus,2,2), sep = ''),' N=',nrow(sp_df),' ',round(max_sim,2),'-',round(min_sim,2), sep='')
    plot(st_geometry(backgroud_map), main = title,
                     col = rgb(.7,.6,.6,.5), xlim = bb_max[c(1,3)], ylim = bb_max[c(2,4)])}
  if (color_scheme > 3){ color_scheme = rgb(0,0,1,.005)}
  
  for (i in 1:length(sp_df$SCINAME)){
    graph_factor = ifelse(i==1,.1,((i-1)/(length(sp_df$SCINAME)-1)))
    map = map_species %>% filter(SCINAME == sp_df$SCINAME[i]) %>% group_by(SCINAME) %>% summarise() %>% st_geometry
    if (color_scheme == 1 | color_scheme == 2 | color_scheme == 3){
      if(color_scheme == 1){color_scheme = rgb(1-graph_factor/1.4,1-graph_factor/2,graph_factor/1.7, graph_factor/2 )
      } else { if(color_scheme == 2) {color_scheme = rgb(1-graph_factor/1.2,graph_factor/1.5,1-graph_factor/1.1, graph_factor/1.8 )
      } else { color_scheme = rgb(graph_factor/1.8,graph_factor/1.3,1-graph_factor/1.7, graph_factor/2 ) }}
    }
    
    plot(map, col = color_scheme, add = T)
  }
  plot(map_species %>% filter(SCINAME == sp_focus) %>%
         group_by(SCINAME) %>% summarise() %>% st_geometry,lwd=3,add=T)
  sp_df
}

# plot_spp_sim_maps -> spp on a vector function 
# {'PLOT SPECIES in a df (SCINAME, dist-sim) and calculates intersection and union as result'
#   'map_species1 = map with all species in a SCINAME col & background_map1 = neotropical geometry'
#   'if plot_level_inter_union the function coherence will plot just the level inter union, not accummulated across levels'
# 'RESULTS in a list with UNION AND INTERSECTION'}
# tab_spp = plot_spp_focus (sp_focus = 'Capito auratus', min_sim = .6, new_window = F, position = FALSE, sim_df = sim_df_lowlands, area_df = area_species) %>% select(SCINAME,dist_sim)}
plot_spp_sim_maps = function (tab_spp = tab_spp,
                              map_species = mapAE_SA,
                              n_levels = FALSE,
                              background_plot = FALSE, # default option to use with coherence_to_sp function!
                              background_map = sa, # if background_map = FALSE no background is ploted
                              title = FALSE, # if title != FALSE needs to be given as character
                              plot_level_inter_union = FALSE,... ) {
    print('this is plot_spp_sim_maps V1')
    tab_spp = tab_spp %>% select('SCINAME'= 1, 'dist_sim'=2) %>% arrange(desc(dist_sim)) %>%
    mutate_if(is.factor,as.character)%>% as.data.frame # not working as tibble!!!
  ## PLOT background
    if (background_plot) {
        if(title != FALSE) {plot (background_map$geometry, main = title, cex.main = 1.3)
        } else {plot (background_map$geometry)}
    }
  
    if (n_levels != FALSE){ group_lvl = ntile(tab_spp$dist_sim,n_levels) }
  
    ## PLOT all species in the level
    for(i in 1 : nrow (tab_spp)) {
        m = map_species %>% filter(SCINAME == tab_spp[i,'SCINAME']) %>% st_geometry
        plot(m, add=T, col=rgb(0,1,0,0.2))
        # intersection # union
        if (i == 1){ inter = m; union = m; next }
        inter_backup = inter
        inter = m %>% st_intersection(inter)
        union = m %>% st_union (union)
        if (st_crs(inter) != st_crs(map_species)) {
          st_crs(inter) = st_crs(map_species);
          st_crs(union) = st_crs(map_species)
        }
        if (length (st_area(inter)) == 0) { inter = inter_backup # keeps the old interesection
        print (paste ('BREAK not all species have commom area -->> ', tab_spp[i,'SCINAME']));
        break }
    }
  # plots outside loop
    print('fim_loop')
    if(plot_level_inter_union){
        # plot (union, add = T, col = rgb (0,0,1,.15), lwd = 2, lty = 1)
        # plot (inter, add = T, lwd = 2, col = rgb (.7,.5,0.2,.8))}#(0,.8,.8,.8)) # the last non-zero interception
        plot(st_geometry(union), add = T, col = rgb(0,1,1,.01), lwd = 2, lty = 1)
        plot(st_geometry(inter), add = T, col = rgb(1,.5,.3,.7), lwd = 2)
    }
list('union' = union, 'intersection' = inter)
}

# plot_one_by_one - graphs comparing sp focus with each species in a table
# 'spp is a data.frame with SCINAME, status_sp_focus and dist_sim columns'
# 'adjust the row_col parameter to the number of total comparisons e.g. 10 maps ~ row_col = c(2,5)'
# 'V9.5 map to map_species'
plot_one_by_one = function (spp = g.ruf_ext, sp_focus = 'Gymnopithys rufigula', map_species = mapAE_SA,
                            row_col = c(2,5), new_window = TRUE, col1 = rgb(0,1,0,.2), col2 = rgb(0,0,1,.2), ...){
  spp %>% nrow %>% print # check number of species and set mfrow
  spp = spp %>% arrange(status_sp_focus,desc(dist_sim))
  if(new_window) {x11(); par(mfrow = row_col)}
  sp_map = map_species %>% filter(SCINAME == sp_focus)
  for(i in 1:nrow(spp)) {
    m = map_species %>% filter(SCINAME == spp$SCINAME[i])
    plot(st_geometry(m),col = col1, main = paste(abbrev(spp$SCINAME[i]), spp$dist_sim[i], spp$status_sp_focus[i]))
    plot(st_geometry(sp_map), col = col2, add=T)
  }}

# 'calculation of inter union ratio after incorporating each species sorted by sim'
# 'results in list with [[df]] [[intersection]] [[union]]; df has the ratio inter_union accumulated and areas inter union for each combination'
inter_union_accum = function (sp_focus, spp_df, map_species = mapAE_SA,
                              plot_map = FALSE, plot_add = TRUE, ...) {
  print('inter_union_accum V1') # 29NOV2018
  df = spp_df %>% arrange(desc(dist_sim))
  sp_focus_map = map_species %>% filter(SCINAME == sp_focus)
  #if(plot_map){ plot(st_geometry(sp_focus_map),col = rgb(0,0,1,.7), main = paste(abbrev(sp_focus),'N=',nrow(df))) }
  intersection = union = sp_focus_map
  df_res = data_frame('IU_accum' = double(), 'area_inter' = double(),'area_union' = double())
  for (i in 1:nrow(df)){
    sp_map = map_species %>% filter(SCINAME == df[i,'SCINAME'])
    intersection = st_intersection(sp_map, intersection)
    union = st_union(sp_map, union)
    intersection_area = st_area(intersection) %>% as.double
    union_area = st_area(union) %>%  as.double
    df_res[i,] = c(round(as.double(intersection_area/union_area),3), as.double(intersection_area), as.double(union_area))
    if(plot_map){  plot(union, col = rgb(0,1,0,1/nrow(df)), add = plot_add)
      plot(intersection, col = rgb(1,0,0,1/nrow(df)), add = plot_add)   
    }
    if(plot_map){ plot(st_geometry(union), lwd = 2.5, add = plot_add)
      plot(st_geometry(sp_focus_map), lwd = 3, add = plot_add)
      plot(st_geometry(intersection), lwd = 2.5, add = plot_add)}
  }
  result = list('df' = cbind(df,df_res), 'intersection' = intersection, 'union' = union)
}

# sugestions to improve this tool ?
# if(examples){
#   test = inter_union_acumm(sp_focus = 'Gymnopithys rufigula', spp_df = g.ruf_int[1:20,], map_species = mapAE_SA, plot_map = TRUE, plot_add = TRUE)
#   test[['df']] }

# 'INTER_PATT - plot & calculate & save shape of intersection and union of a group of species'
# 'used in sp_levels_sim for species in the "level"'
# 'depends on "bb_max_spp"'
inter_patt = function (sp_df = species_df, sort_by_sim = FALSE, title = 'title', map_species = mapAE_SA, plot_background = TRUE, new_window = FALSE, background_map = sa,... ){
    # print('inter_patt V1.1 29NOV2018') # option NO plot; mapSA internal was exchanged to call object 'map_species' using mapAE_SA in default
    print('inter_patt V1.2 17MAI2019') # map_species in bb_max_spp call
    break_function = FALSE
    
    bb_max_spp_lvl = bb_max_spp (tab_spp = sp_df, column_spp = 'SCINAME', map_base = map_species)
    
    # check for NAs caused by spelling
    if(bb_max_spp_lvl %>% is.na %>% any) {
        sp_df[,column_spp]  = sp_df[,column_spp] %>% gsub('_', ' ',.)
        bb_max_spp_lvl = bb_max_spp (tab_spp = sp_df, column_spp = 'SCINAME', map_base = map_species)
    }
    # check again changing space to underline
    if(bb_max_spp_lvl %>% is.na %>% any) {
        sp_df[,column_spp]  = sp_df[,column_spp] %>% gsub(' ', '_',.)
        bb_max_spp_lvl = bb_max_spp (tab_spp = sp_df, column_spp = 'SCINAME', map_base = map_species)
    }
    
    if (sort_by_sim == TRUE & 'dist_sim' %in% names(sp_df)) { sp_df = sp_df %>% arrange(desc(dist_sim)) }
    map_1 = map_species %>% filter(SCINAME == sp_df[1,'SCINAME']) %>% st_geometry
    
    if(plot_background){
        if(new_window) { x11() }                #; par(mar=c(1,3,1,1)) }
        plot(background_map$geometry, xlim = bb_max_spp_lvl[c(1,3)], ylim = bb_max_spp_lvl[c(2,4)], col=rgb(0,.3,0,.4))
        title(main = title, cex.main = 1.5)
    }
    plot(map_1, add = T, col = rgb(1,0,0,.1))
    
    for(i in 2:nrow(sp_df)){
        m = map_species %>% filter(SCINAME == sp_df[i,'SCINAME']) %>% st_geometry
        plot(m, add = T, col = rgb(0,0,1,0.1))
        if (i ==2) {union = m %>% st_union(map_1)} else { union = m %>% st_union(union) %>% st_geometry() }
        if (i ==2) {inter = m %>% st_intersection(map_1) %>% st_geometry} else { inter = m %>% st_intersection(inter) %>% st_geometry }
        
        if (length(st_area(inter)) == 0) {
            print('not all species have commom area')  # not sure if this break is working
            print(paste(sp_df[i,'SCINAME'], 'in', sp_df[,'SCINAME']))
            break_function = T
            break }
    }
    if(break_function) {print('No intersection till the end')
        plot(background_map$geometry, xlim = bb_max_spp_lvl[c(1,3)], ylim = bb_max_spp_lvl[c(2,4)], col=rgb(0,.3,0,.4))
        break }
        plot(inter, add = T, col = rgb(0,1,1,1),lwd = 1.4) # red the last non-zero interception
    plot(union,add = T, col = rgb(.4,1,1,.02), lwd = 2)
        maps = list()
    maps[['intersect']] = inter  # the returned object is the intersection sf shape
    maps[['union']] = union
    
    maps      # list with intersection and union 
}

#### Auxiliary tools ####
# ex-tab_spp wraper to make fast tab_spp tables [,c(SCINAME, dist_sim)]
sim_sp_df = function (sp_focus, max_sim = 1, min_sim = .7, similarity_df = sim_df) {
  similarity_df %>% filter(species1 == sp_focus) %>% filter (species2!= sp_focus) %>% 
  select('SCINAME' = species2, dist_sim) %>% filter(dist_sim >= min_sim & dist_sim <= max_sim) %>%
  mutate_if(is.factor, as.character) %>% arrange(desc(dist_sim)) %>% as.data.frame()
  }

# BB MAX SPECIES 
'Calculate the box of max-min geographical coordinates for a vector of species or data frame'
bb_max_spp = function (tab_spp = tab_spp, column_spp = 'SCINAME', map_base = mapAE_SA) { 
    print('bb_max_spp V1.1') # tab_spp is a df with species in a column SCINAME; map_base object as a parameter
    box_df = data_frame ()
    if (is.vector(tab_spp)){ tab_spp = data.frame( 'SCINAME' = tab_spp, stringsAsFactors=F)} # check column_spp, if matches the name, ok
    # tab_spp = tab_spp %>% mutate_if(is.factor,as.character)
    for (i in 1:nrow(tab_spp)){
        # map & data frame with all boxes
        map1 = map_base %>% filter(SCINAME == tab_spp[i,column_spp]) %>% st_geometry
        box_df = rbind(box_df,data_frame('xmin'= st_bbox(map1)[1],'ymin'= st_bbox(map1)[2],
                                         'xmax'= st_bbox(map1)[3],'ymax'=st_bbox(map1)[4] ))
    }
    #check spelling '_' vs. ' '
    if ( box_df %>% is.na %>% all) { 
        box_df = data_frame ()
        tab_spp[,column_spp] = tab_spp[,column_spp] %>% gsub('_', ' ',.)
    for (i in 1:nrow(tab_spp)){
        # map & data frame with all boxes
        map1 = map_base %>% filter(SCINAME == tab_spp[i,column_spp]) %>% st_geometry
        box_df = rbind(box_df,data_frame('xmin'= st_bbox(map1)[1],'ymin'= st_bbox(map1)[2],
                                         'xmax'= st_bbox(map1)[3],'ymax'=st_bbox(map1)[4] ))
    }    }
    
    xy = c('xmin'=min(box_df$xmin), 'ymin'=min(box_df$ymin), 'xmax'=max(box_df$xmax), 'ymax'=max(box_df$ymax))
    xy
}
if(examples) { 
  bb_max_test = bb_max_spp(tab_spp, map_base = mapAE_SA)}

# check for shared species between lots of species in a pattern against a list of species patterns
check_spp_patt = function(sp_patt, spp_chk, thr_df = thr, only_name_number = T) {
    for (sp in spp_chk){
        sp_patt_list = thr_df %>% filter(species1 == sp_patt) %>% group_by(species1,species2) %>%
            summarise(n = n(), sim = mean(dist_sim), min = min(min), max = max(max)) %>%
            group_by(species2, n, sim, min, max) %>% summarise()
        df = thr_df %>% filter(species1 == sp ) %>% group_by(species1,species2) %>% 
            summarise(n = n(), sim = mean(dist_sim), min = min(min), max = max(max)) %>% 
            group_by(species2, n, sim, min,max) %>% summarise()    
        n = inner_join(df, sp_patt_list, by = c('species2'))
        if(only_name_number) { print(paste(sp_patt,'    x     ',sp,'   :',  nrow(n), ' spp  ', ifelse(nrow(n)==0,'...ZERO!!!!','')))} else {print(n)}
    }
}

# save shapefiles of all species of a complex  USES THR data which is 
# thr is the output of threshold_levels function | the map needs to have a SCINAME column for species names
cplx_to_shapefile = function(sp_focus = sp_focus, threshold_table = thr, mapAE_SA = map_species_lowland_50, ...){
    shp_cplx = mapAE_SA %>% filter( SCINAME == sp_focus | SCINAME %in% thr[which(thr$species1==sp_focus),]$species2 )
    st_write (shp_cplx,   update=TRUE,   dsn = getwd(),   layer =  paste(gsub(' ','_',sp_focus), '_cplx', sep='') ,    driver = 'ESRI shapefile')
    print(paste('shapes of the ', sp_focus, ' biotic complex saved: ', paste(getwd(), '/',paste(gsub(' ','_',sp_focus), '.shp', sep=''), sep='')))
}

#little functions to display taxa in an element using the reference species (sp1fun)
#and all elements a species is composing (sp2fun)
sp1fun = function(sp_focus){sp1_sp2 %>% filter(species1==sp_focus)}
sp2fun = function(sp_focus){sp1_sp2 %>% filter(species2==sp_focus)}

# wrap to make fast list of sim_df 
list_df = function (sp_focus, max_sim, min_sim, df_sim = sim_df){
  print('list_df V2')# option to sp_focus
  if(exists('sp_focus')){
    df = df_sim %>% filter(species1 == sp_focus) %>% filter(species1 != species2) %>%
    filter (dist_sim <= max_sim & dist_sim >= min_sim) %>% arrange(desc(dist_sim))
  } else {
   df = df_sim %>% filter(species1 != species2) %>%
        filter (dist_sim <= max_sim & dist_sim >= min_sim) %>% arrange(desc(dist_sim))}
  df
  }

# abbreviate species names
abbrev = function(x){paste(str_sub(x,1,1),'.', word(x,2,2), sep = '')}

# https://stackoverflow.com/questions/47749078/how-to-put-a-geom-sf-produced-map-on-top-of-a-ggmap-produced-raster
# Define a function to fix the bbox to be in EPSG:3857
ggmap_bbox <- function(map) {
    if (!inherits(map, "ggmap")) stop("map must be a ggmap object")
    # Extract the bounding box (in lat/lon) from the ggmap to a numeric vector, 
    # and set the names to what sf::st_bbox expects:
    map_bbox <- setNames(unlist(attr(map, "bb")), 
                         c("ymin", "xmin", "ymax", "xmax"))
    
    # Coonvert the bbox to an sf polygon, transform it to 3857, 
    # and convert back to a bbox (convoluted, but it works)
    bbox_3857 <- st_bbox(st_transform(st_as_sfc(st_bbox(map_bbox, crs = 4326)), 3857))
    
    # Overwrite the bbox of the ggmap object with the transformed coordinates 
    attr(map, "bb")$ll.lat <- bbox_3857["ymin"]
    attr(map, "bb")$ll.lon <- bbox_3857["xmin"]
    attr(map, "bb")$ur.lat <- bbox_3857["ymax"]
    attr(map, "bb")$ur.lon <- bbox_3857["xmax"]
    map
}

# Check overlap between species and whole patterns (sp_patt is a vector with one spp; spp_chk vector with many spp)
# uses thr data frame resulting from a 'threshold-levels' tool
 check_spp_patt = function(sp_patt, spp_chk, thr_df = thr, only_name_number = T) {
    for (sp in spp_chk){
        sp_patt_list = thr_df %>% filter(species1 == sp_patt) %>% group_by(species1,species2) %>%
            summarise(n = n(), sim = mean(dist_sim), min = min(min), max = max(max)) %>%
            group_by(species2, n, sim, min, max) %>% summarise()
        df = thr_df %>% filter(species1 == sp ) %>% group_by(species1,species2) %>% 
            summarise(n = n(), sim = mean(dist_sim), min = min(min), max = max(max)) %>% 
            group_by(species2, n, sim, min,max) %>% summarise()    
        n = inner_join(df, sp_patt_list, by = c('species2'))
        if(only_name_number) { print(paste(sp_patt,'    x     ',sp,'   :',  nrow(n), ' spp  ', ifelse(nrow(n)==0,'...ZERO!!!!','')))} else {print(n)}
    }
}

## THIS VERSION WORKS AFTER source_clean_V9.5
# Check overlap in species from patterns (sp_patt is a vector with one spp; spp_chk vector with many spp)
# uses thr1 data frame for varzea data frame structure (species1,species2,Csim,Cthres_max,Cthres_min, depth_min, depth_max)
check_spp_patt_2 = function(sp_patt, spp_chk, thr_df = thr, only_name_number = T) {
     results_df = data_frame()
     for (sp in spp_chk){
         sp_patt_list = thr_df %>% filter(species1 == sp_patt) %>% group_by(species1,species2) %>%
             summarise(n = n(), Cs_mean = mean(Csim), Ct_min = min(Cthres_min), Ct_max = max(Cthres_max)) %>%
             group_by(species2, Cs_mean, Ct_min, Ct_max) %>% summarise(n())
         
         df = thr_df %>% filter(species1 == sp ) %>% group_by(species1,species2) %>% 
             summarise(n = n(), Cs_mean = mean(Csim), Ct_min = min(Cthres_min), Ct_max = max(Cthres_max)) %>% 
             group_by(species2, Cs_mean, Ct_min, Ct_max) %>% summarise()
         
         n = inner_join(df, sp_patt_list, by = c('species2')) %>% select(common_spp = species2, everything())
         N_spp_patt = thr_df %>% filter(species1 == sp_patt) %>% group_by(species1,species2) %>% nrow()
         N_spp_chk = thr_df %>% filter(species1 == sp) %>% group_by(species1,species2) %>% nrow()
         if(only_name_number) { 
             # print(paste(sp_patt, '(',N_spp_patt,'spp)', '    ',  nrow(n), ' spp', '     ', sp, '(',N_spp_chk,'spp)', ifelse(nrow(n)==0,'...ZERO!!!!','')))
             results_df = rbind(results_df, data_frame(species1 = sp_patt, nspp1 = N_spp_patt, common_spp = nrow(n), nspp2 = N_spp_chk, species2 = sp))%>%
                 arrange(desc(common_spp), desc(nspp2))
             } else {print(sp); print(n)}
     }
results_df
}

# PLOT PATTERNS
# backgroup with Areas of Endemism for Amazonia
# # plot species focus and its pattern species2 in sp1_sp2 data frame or a list of species inside a vector >1 length.
# ggmap_bbox function is used to convert the bbox of the ggmap image to crs compatible (3857...) everything is 3857
# to convert use the line 'st_bbox(st_transform(st_as_sfc(st_bbox(OBJECT_box, crs = 4326)), 3857))'
# 
ggpatt = function(sp_focus, df = sp1_sp2, mapa = mapAE_SA, bg_map = sa_ggmap_z4, ae_map = mapAE_SA[1:9,], plot_ae = FALSE,
                  bbox_lim = T, # buffer = 1.5,
                  new_window=FALSE, title = paste(sp_focus), alph = .05, line_wdt = 0.5, rgb_colors = 'dark green',...){
    print('this is ggpat V1.1') # V1.1 with ae shp & vector with spp option
    # make a map with all species 
    if(length(sp_focus) == 1) { lista = df %>% filter(species1 == sp_focus) %>% select(species2) 
    } else { lista = data_frame('species2' = sp_focus) 
            print('ploting only species in the vector "sp_focus", not a pattern') }
    # map
    ggplatt = ggmap(ggmap_bbox(bg_map)) + ggtitle(title) + theme(plot.title = element_text(size = 40, face = "bold"))
    
    if(bbox_lim){ 
        bbox_species = bb_max_spp(tab_spp = lista$species2, map_base = mapa)
        # bbox_species = st_buffer(st_as_sfc(st_bbox(bbox_species, crs = 4326)), buffer) %>% attr(.,'bbox') # buffer = 1.5 degrees
        # can't make the buffer work... I will try again in another opportunity...
        # convert bbox_species to 
        bbox_species = st_bbox(st_transform(st_as_sfc(st_bbox(bbox_species, crs = 4326)), 3857))
        ggplatt = ggplatt + xlim (bbox_species[['xmin']], bbox_species[['xmax']]) + ylim (bbox_species[['ymin']], bbox_species[['ymax']])  }
    
    if(plot_ae) {  # map with areas of endemism or whatever natural or political shapefile object
        ggplatt =  ggplatt + geom_sf(data = st_geometry(st_transform(fortify(ae_map),crs = 3857)),
                        aes(),lwd = .5, lty = 1, alpha = 0.01, col = 'black', inherit.aes = FALSE) }
    
    if(length(sp_focus) == 1) {
        map_species1 = 
            mapa %>%
            filter(SCINAME == sp_focus) %>%
            select(species2 = SCINAME) %>%
            st_transform(crs = 3857) %>% 
            fortify()
    } else { 
        map_species1 =
            mapa %>%
            filter(SCINAME == lista$species2[1]) %>%
            select(species2 = SCINAME) %>%
            st_transform(crs = 3857) %>%
            fortify()
    }
    
    ggplatt = ggplatt + geom_sf(data = st_geometry(map_species1), aes(), alpha = alph, col = 'black', lwd = line_wdt, inherit.aes = FALSE) 
    
    for(n in 1: nrow(lista)){
        sp = as.character(lista[n,'species2'])
        map_species2 = mapa %>% filter(SCINAME == sp) %>% select(species2 = SCINAME) %>% st_transform(crs = 3857)
        
        # load all maps at ggplatt object
        ggplatt = ggplatt + geom_sf(data = map_species2, aes(), alpha = alph, lwd = .8, lty = 1, fill = rgb_colors, col = 'black', inherit.aes = FALSE)
    }
    
    if(new_window) x11()
    print(lista)
    ggplatt
}

if(examples){
    sp1_sp2 %>% filter(word(species1,1)=='Rhegmatorhina') %>% group_by(species1) %>% summarise %>% .$species1 -> spp
    test = ggpatt(sp_focus=spp, plot_ae = T);    test
}

#__________________________________________________________________________#
# ae_sa = sa %>% select(group) %>% rbind(.,(ae %>% select(group = SCINAME)))
plot_patt = function(sp_focus, df = sp1_sp2, map = mapAE_SA, ae_map = sa_ae, add_map = FALSE,
                     red = .5, green = .5, blue = .5, alpha = .3,...){
    if (!add_map){plot(st_geometry(ae_map[1,]),col=rgb(.2,1,.2,.3))
        plot(st_geometry(ae_map[2:10,]),col=rgb(.1,.8,.2,.2),lwd=1.3,add=T)}
    for(sp in (df %>% filter(species1== sp_focus) %>% select(species2))){
        map %>% filter(SCINAME == sp) %>% st_geometry %>% plot(add=T,col=rgb(red,green,blue,alpha),lwd=1.3)}
}
#______________________________________________________________

# print('This is source_clean_V9.5 
print('This is congruence_source_1.1.R')
