# Supplementary material for: Spatial Congruence Analysis (SCAN): A method for detecting biogeographical patterns based on species range congruences
Source: PLoS One. 2021 May 20;16(5):e0245818. doi: 10.1371/journal.pone.0245818 (PMC8136640; doi:10.1371/journal.pone.0245818)
Supplement: S2 Script — The tutorial and the source script are better viewed in a code editor, such as Rstudio (www.rstudio.org). The functions presented at the Tutorial S2.1 are running and integrated, but the whole framework is still in early stages of code development. Some functions are auxiliary tools, such as coherence_to_sp, which uses congruence and depth to plot range relationships in a customized way (indicating ‘internal’, ‘external’, and other spatial relations). Many others are intermediary tools called by higher hierarchical functions. New versions will maintain the functionality but the code will be refined, re-organized as a package, and uploaded opportunistically at https://github.com/cassianogatto/congruence_source (current is congruence_source_1.1.R). (RTF) [file pone.0245818.s009.rtf]

Spatial Congruence Analysis (SCAN): A method for detecting biogeographical patterns based on species’ range congruences
Cassiano AFR Gatto & Mario Cohn-Haft

S2 Script. A brief tutorial to apply the congruence framework to the theoretical simulated
 gradient of ranges of Kreft & Jetz (2013) [34]⁠. The tutorial and the source script are better viewed in a code editor, such as Rstudio (www.rstudio.org). The functions presented at the Tutorial S1.1 are running and integrated, but the whole framework is still in early stages of code development. Some functions are auxiliary tools, such as coherence_to_sp, which uses congruence and depth to plot range relationships in a customized way (indicating ‘internal’, ‘external’, and other spatial relations). Many others are intermediary tools called by higher hierarchical functions. New versions will maintain the functionality but the code will be refined, re-organized as a package, and uploaded opportunistically at https://github.com/cassianogatto/congruence_source (current is congruence_source_1.1.R).

#### Implementation of Kreft & Jetz 2013 grdient in R ####

setwd ('C:/SIG2018/kreft_jetz_example') # choose your directory folder where source is saved

source('congruence_source_1.1.R')

# create polygons
{s1 = st_polygon(list(rbind(c(0,0),c(0,1),c(1,1),c(1,0),c(0,0))))
s2 = st_polygon(list(rbind(c(0,0),c(0,1),c(2,1),c(2,0),c(0,0))))
s3 = st_polygon(list(rbind(c(0,0),c(0,1),c(4,1),c(4,0),c(0,0))))
s4 = st_polygon(list(rbind(c(0,0),c(0,1),c(5,1),c(5,0),c(0,0))))
s5 = st_polygon(list(rbind(c(0,0),c(0,1),c(6,1),c(6,0),c(0,0))))
s6 = st_polygon(list(rbind(c(0,0),c(0,1),c(7,1),c(7,0),c(0,0))))
s7 = st_polygon(list(rbind(c(0,0),c(0,1),c(8,1),c(8,0),c(0,0))))
s8 = st_polygon(list(rbind(c(0,0),c(0,1),c(9,1),c(9,0),c(0,0))))
s9 = st_polygon(list(rbind(c(0,0),c(0,1),c(10,1),c(10,0),c(0,0))))
s10 = st_polygon(list(rbind(c(0,0),c(0,1),c(11,1),c(11,0),c(0,0))))
s11 = st_polygon(list(rbind(c(0,0),c(0,1),c(13,1),c(13,0),c(0,0))))
s12 = st_polygon(list(rbind(c(0,0),c(0,1),c(15,1),c(15,0),c(0,0))))
s13 = st_polygon(list(rbind(c(0,0),c(0,1),c(16,1),c(16,0),c(0,0))))
s14 = st_polygon(list(rbind(c(0,0),c(0,1),c(17,1),c(17,0),c(0,0))))
s15 = st_polygon(list(rbind(c(0,0),c(0,1),c(18,1),c(18,0),c(0,0))))
n16 = st_polygon(list(rbind(c(30,0),c(12,0),c(12,1),c(30,1),c(30,0))))
n17 = st_polygon(list(rbind(c(30,0),c(13,0),c(13,1),c(30,1),c(30,0))))
n18 = st_polygon(list(rbind(c(30,0),c(14,0),c(14,1),c(30,1),c(30,0))))
n19 = st_polygon(list(rbind(c(30,0),c(15,0),c(15,1),c(30,1),c(30,0))))
n20 = st_polygon(list(rbind(c(30,0),c(17,0),c(17,1),c(30,1),c(30,0))))
n21 = st_polygon(list(rbind(c(30,0),c(19,0),c(19,1),c(30,1),c(30,0))))
n22 = st_polygon(list(rbind(c(30,0),c(20,0),c(20,1),c(30,1),c(30,0))))
n23 = st_polygon(list(rbind(c(30,0),c(21,0),c(21,1),c(30,1),c(30,0))))
n24 = st_polygon(list(rbind(c(30,0),c(22,0),c(22,1),c(30,1),c(30,0))))
n25 = st_polygon(list(rbind(c(30,0),c(23,0),c(23,1),c(30,1),c(30,0))))
n26 = st_polygon(list(rbind(c(30,0),c(24,0),c(24,1),c(30,1),c(30,0))))
n27 = st_polygon(list(rbind(c(30,0),c(25,0),c(25,1),c(30,1),c(30,0))))
# n28 = st_polygon(list(rbind(c(30,0),c(26,0),c(26,1),c(30,1),c(30,0))))
n28 = st_polygon(list(rbind(c(30,0),c(27.5,0),c(27.5,1),c(30,1),c(30,0)))) # mod
n29 = st_polygon(list(rbind(c(30,0),c(28,0),c(28,1),c(30,1),c(30,0)))) # mod
# n30 = st_polygon(list(rbind(c(30,0),c(29,0),c(29,1),c(30,1),c(30,0))))
n30 = st_polygon(list(rbind(c(30,0),c(28.5,0),c(28.5,1),c(30,1),c(30,0)))) # mod

d = data.frame(region = c(rep('S',15),rep('N', 15)),id = (1:30))
d$geometry = st_sfc(s1,s2,s3,s4,s5,s6,s7,s8,s9,s10,s11,s12,s13,s14,s15,n16,n17,n18,n19,n20,n21,n22,n23,n24,n25,n26,n27,n28,n29,n30)
pol = st_as_sf(d)
pol %>% st_is_valid %>% all()
pol %>% st_set_geometry(NULL) %>% group_by(region, id) %>% summarize
}
# polygons = pol %>% group_by(region) %>% summarise_all(first) %>% st_cast('MULTIPOLYGON') # this seems to merge the polygons
# plot((polygons %>% st_geometry)+c(0,2),col = 'green', add = T)
# plot species' multipolygons (not sf objects yet)
dev.new(); par(mfrow=c(2,1))
plot(c(),axes=T,xlim=c(0,31),ylim=c(-2,2)) # blank template
plot(pol[15:1,] %>% st_geometry, col = c(rgb(0,0,1,.2)),add = T)
plot((pol[16:30,] %>% st_geometry) - c(0,1) , col = c(rgb(1,0,0,.2)), add = T)
plot('.',axes=T,xlim=c(0,31),ylim=c(-15,15))# blank template
for (i in 1:15){
    plot(pol[i,] %>% st_geometry + c(0,(15-i)), add = T, col='blue')
    plot(pol[31-i,] %>% st_geometry + c(0,(-16+i)), add = T, col = 'red')
}

## overlap and Cs table##
{overlap_row = st_intersection(pol, pol)
area = st_area(overlap_row)
overlap = overlap_row %>% st_set_geometry(NULL) %>% cbind(area) %>% mutate( p1 = paste(region, as.character(id), sep=''), p2 = paste
                                                (region.1, as.character(id.1),sep='')) %>% select(p1,p2,area_overlap = area, everything())
area_pol = pol %>% st_set_geometry(NULL) %>% mutate(p = paste(region, as.character(id),sep='')) %>% cbind(data.frame('area_pol' = st_area(pol)))

Cs = left_join(overlap, area_pol, by = c('p1'='p')) %>% mutate(area_p1 = area_pol, reg_p1 = region.x,id_p1 = id.x, reg_p2 = region.1, id_p2 = id.1) %>% 
    select(p1,p2,area_overlap, area_p1,reg_p1,id_p1,reg_p2,id_p2)
Cs = Cs %>% left_join(area_pol, by = c('p2' = 'p')) %>% select(p1,p2,area_overlap, area_p1,area_p2 = area_pol, everything())
Cs = Cs %>% mutate(Cs = (area_overlap/area_p1)*(area_overlap/area_p2)) %>% select(p1,p2,Cs,area_overlap, area_p1,area_p2,r1 = reg_p1,i1 = id_p1,r2 =reg_p2,i2=id_p2)
Csbkp = Cs
Cs = Cs %>% filter(p1 != p2) %>% filter(Cs > 0)
Cs$dist_sim = Cs$Cs; Cs$species1 = Cs$p1; Cs$species2 = Cs$p2}

# check table
Cs %>% head

#### threshold analysis #### 
# list of species and (SETUP objects for debug)
pol = pol %>% mutate(SCINAME = paste(region, id,sep=''))
list_spp = data_frame('SCINAME' = unique(pol$SCINAME)) 
# map_species = pol;1; min_sim_init=.95; sim_df = Cs; n_levels = 7

#### Threshold Levels ####
{threshold_level3 =  threshold_levels(spp_df = list_spp, map_species = pol, min_sim_init=.95, sim_df = Cs, n_levels = 3)
threshold_level5 =  threshold_levels(spp_df = list_spp, map_species = pol, min_sim_init=.95, sim_df = Cs, n_levels = 5)
threshold_level7 =  threshold_levels(spp_df = list_spp, map_species = pol, min_sim_init=.95, sim_df = Cs, n_levels = 7)
threshold_level10 =  threshold_levels(spp_df = list_spp, map_species = pol, min_sim_init=.95, sim_df = Cs, n_levels = 10)
all.equal(names(threshold_level3),names(threshold_level5))}
#build a unified table thr_all
thr_all = rbind(threshold_level3 %>% mutate(max_depth_set = 3),threshold_level5 %>% mutate(max_depth_set = 5),
                threshold_level7 %>% mutate(max_depth_set = 7),threshold_level10 %>% mutate(max_depth_set = 10))

#### Biotic Elements - summary ####
 
thr = thr_all %>% left_join(Cs) %>% select(max_depth_set, species1,species2,Csim = Cs, Cthres, depth, status, area_overlap, area_p1, area_p2)
# NON-OVERLAPPING CLOSED GROUPS
non_overlap = thr %>% group_by(max_depth_set, species1,Cthres,status) %>% summarise(Cs_mean = mean(Csim),spp_group = paste(species2, collapse = ', ')) %>% arrange(max_depth_set,parse_number(species1),desc(Cthres)) %>% filter(is.na(Cs_mean))
# below, all closed groups with any member not overlapping any other are excluded
biotic_elements = {thr %>% group_by(max_depth_set, species1,Cthres,status) %>% 
    summarise(Cs_mean = mean(Csim),depth = max(depth),
                spp_group = sapply(strsplit(paste(species2, collapse = ', '),', '), 
                                   function(x) paste(unique(x)[order(parse_number(unique(x)))], collapse = ', '))) %>% 
                mutate(n_spp = sapply(spp_group, function(x) {strsplit(x,', ') %>% unlist %>% length})) %>%
                filter(!is.na(Cs_mean)) %>%
                select(max_depth_set, species1, Cthres, status, n_spp, Cs_mean, depth, spp_group) %>% 
                arrange(max_depth_set,parse_number(species1),desc(Cthres))}
# all biotic elements RAW DATA
biotic_summary = {biotic_elements %>% group_by(max_depth_set,species1,Cs_mean,n_spp,spp_group) %>% 
    summarise(Ctmax = max(Cthres),Ctmin = min(Cthres), depth = max(depth)) %>% 
    select(1,2,6,7,4,3,8,5) %>% arrange(max_depth_set, parse_number(species1), desc(Ctmax,n_spp))}
biotic_summary
# summarised results (table by depth) 
thr_lite = {biotic_elements %>% group_by(max_depth_set,species1) %>% summarise(Ct_max = max(Cthres), Ct_min = min(Cthres), 
                        Cs_mean = mean(Cs_mean), depth_max = max(depth), depth_min=min(depth), 
                        spp_group = sapply(strsplit(paste(spp_group, collapse = ', '),', '),
                                           function(x) paste(unique(x)[order(parse_number(unique(x)))], collapse = ', '))) %>% # sapply to break each string (each closed group) and paste unique values to string
                        mutate(n_spp = sapply(spp_group, function(x) {strsplit(x,', ') %>% unlist %>% length})) %>%   # sapply to break strings and count (lenght)
                        select(max_depth_set, species1,n_spp, Cs_mean, Ct_max, Ct_min, depth_max, depth_min, spp_group) %>%
                        arrange(max_depth_set, parse_number(species1), desc(Ct_max,n_spp))} # thr_lite %>% write.table('clipboard')
thr_lite
# super summarized by species and max depth (except for max depth = 10 -> all max groups converge to all overlapping species)
biotic_summary_lite = {biotic_summary %>% 
    filter(max_depth_set != 10) %>% group_by(species1) %>%
        summarise(max_depth_set = paste(unique(max_depth_set), collapse = ', '),
        Ctmax = max(Ctmax), Ctmin = min(Ctmin), nsppmax = max(n_spp),nsppmin = min(n_spp),
        Csmean = mean(Cs_mean), depthmax = max(depth), depthmin = min(depth),
        spp_all = sapply(strsplit(paste(spp_group, collapse = ', '),', '),
            function(x) paste(unique(x)[order(parse_number(unique(x)))], collapse = ', '))) %>%
        arrange(parse_number(species1))}
biotic_summary_lite
# unique species combinations in biotic elements
unique_biotic = {biotic_summary %>% mutate(species_biotic = paste(species1,spp_group,sep=', ')) %>% 
                mutate(species_biotic = sapply(strsplit(paste(species_biotic, collapse = ', '),', '), 
                                        function(x) paste(unique(x)[order(parse_number(unique(x)))], collapse = ', ')))}

# unique_biotic_elements = {unique_biotic  %>%  group_by(species_biotic) %>% summarize(sp1 = sapply(strsplit(paste(species1, collapse = ', '),', '), 
#                                         function(x) paste(unique(x)[order(parse_number(unique(x)))], collapse = ', ')),
#                                         depth_max = sapply(strsplit(paste(max_depth_set, collapse = ', '),', '), 
#                                                    function(x) paste(unique(x)[order(parse_number(unique(x)))], collapse = ', '))) %>% 
#                         arrange(parse_number(species_biotic)) %>% as.data.frame}

unique_biotic_elements_357depth = {unique_biotic %>% filter(max_depth_set != 10)  %>%  group_by(species_biotic) %>%
                                summarize(Ctmax = round(mean(Ctmax),2), sp1 = sapply(strsplit(paste(species1, collapse = ', '),', '), 
                                    function(x) paste(unique(x)[order(parse_number(unique(x)))], collapse = ', ')),
                                     depth_max_set = sapply(strsplit(paste(max_depth_set, collapse = ', '),', '), 
                                      function(x) paste(unique(x)[order(parse_number(unique(x)))], collapse = ', '))) %>% 
                arrange(parse_number(species_biotic)) %>% select(3,2,1) %>% arrange(sp1,species_biotic) %>% as.data.frame}

#### Synonyms ####
## # this section can be used to select species and thresholds to analyze and compare to other reference species. It can be used to draw the diagrams - 
## first select the reference species (sp_focus) and the congruence threshold (cong). The mm2 table is a very refined summary for this species
## relationships at this congruence threshold. 'sp_levels_sim' is used to draw the customized graphics
tab = thr
sp_focus = 'S8'  # with S3 I got an error below - it does not generate biotic elements (I need to fix this bug with empty table from sp_levels_sim -> threshold_levels)

mm = threshold_levels(spp_df = data_frame(SCINAME= c('S8')), min_sim_init=.95, sim_df = Cs, n_levels=5)
mm = mm %>% left_join(., Cs) %>% mutate(Csim = Cs) %>% select(species1,species2,Cthres,Csim,depth,status,area_overlap,area_p1,area_p2)
mm1 = mm %>% group_by(species1,species2, Csim, Cthres) %>% summarize(depth = min(depth) %>% as.integer()) %>% arrange(desc(Cthres),depth,desc(Csim));mm1 %>% as.data.frame
mm2 = mm1 %>% group_by(species1,species2,Csim) %>% summarise(Ct_max = round(max(Cthres),2), Ct_min = round(min(Cthres),2), depth = max(depth)) %>%
    select(species1,species2,Ct_max, Ct_min, depth, Csim) %>% mutate(C_sim = round(Csim,2)) %>% arrange(desc(Ct_max), depth, desc(Csim)) %>% as.data.frame; mm2
# mm2 # this is the profile for the species at this max_depth set (n_levels = 5)
# species1 species2 Ct_max Ct_min depth      Csim C_sim
# 1       S8       S9   0.90   0.85     1 0.9000000  0.90
# 2       S8      S10   0.90   0.85     2 0.8181818  0.82
# 3       S8       S7   0.88   0.85     1 0.8888889  0.89
# 4       S8       S6   0.87   0.85     2 0.7777778  0.78
# 5       S8       S5   0.85   0.85     3 0.6666667  0.67

# setup Cs  - choose the CONGRUENCE THRESHOLD you want to be ploted or analyzed!!!
cong = 0.51 #'(cong is Cs but there is already and object table Cs)'
par(mfrow=c(5,4))

assign(paste(sp_focus,'_mm', cong, sep = ''), sp_levels_sim (sp_focus = sp_focus, map_species = pol, background = st_union(pol) %>% st_geometry, plot_background = TRUE, # did not work without background map - I used st_union
           min_sim = cong, sim_df = Cs,  n_levels =cong, abbrev = F) ) # higher  level 7 species

#### I/U extraction from intersection and union polygons ####
# recovering I/U data from intersection/union polygons generated at threshold analyses (not used here for Kreft & Jet 2013 example)
lev = 1
IU = get(paste(sp_focus,'_mm', cong, sep = ''))['inter_union_ratio'][[1]] %>% as.numeric %>% round(.,2)
#save IU at this Cthres for this species
assign(paste(sp_focus,'_IU_', cong, sep = ''), get(paste(sp_focus,'_mm', cong, sep = ''))['inter_union_ratio'])
# call fot Intersection/union ratio
get(paste(sp_focus,'_IU_', cong, sep = ''))
# ll = get(paste(sp_focus,'_mm', cong, sep = ''))[[paste('level', lev, sep = '')]]
# inter_union = inter_patt(ll, map_species = pol, title = paste (sp_focus, '\n', cong, '    level', lev, '    ', nrow(ll),'spp', sep=''),
#                          background_map = st_union(st_geometry(pol)))

# ####################################### #
#### GRAPHS SPECIES BIOTIC ELEMENTS ####
# ####################################### #
# check Cthres
# for species N29 at  max depth = 7 thre range of congruence thresholds generating biotic elements
thr_all %>% filter(species1=='N29'& max_depth_set == 7) %>% summarise(range(Cthres)[1], range(Cthres)[2])

#### run sp_levels_sim graphics
dev.new()
par(mfrow = c(6,5), mar = c(3,2,4,2))
# for(sp_focus in (c('S1','S6','S8','S11','S15','N17','N21','N24','N29'))){
    # error = character()
    # sp_run = 
        try(sp_levels_sim (sp_focus = 'S12', map_species = pol,
               background = st_union(pol) %>% st_geometry, plot_background = TRUE, # did not work without background map - st_union is a nice workaroundf
               min_sim = 0.87, sim_df = Cs, 
               n_levels =7))
    # if ('try-error' %in% class(sp_run)) {error = paste(error,sp_focus); next}
    # print(error)
# }
#### PLOTs ####
#### Congruencegrams  # see 
par(mfrow = c(6,5), mar = c(3,2,4,2))
# PLOT species S12
biotic_summary %>% filter(species1=='S12' & max_depth_set ==10) %>% as.data.frame
biotic_summary %>% filter(species1=='S12' & max_depth_set ==10) %>% group_by(spp_group) %>% summarise(minCT=min(Ctmin)) %>% arrange(desc(minCT))              

for (Ct in c(.87,.85,.84,.81,.51,.12)){
    try(sp_levels_sim (sp_focus = 'S12', map_species = pol,
                       background = st_union(pol) %>% st_geometry, plot_background = TRUE, # did not work without background map - st_union is a nice workaroundf
                       min_sim = Ct, sim_df = Cs, 
                       n_levels =10))}
   # # horizontal base
base_horiz = function( use_title = FALSE, title = '', xlim = c(0,31), ylim = c(0,3)) {
    if(use_title) {plot(pol[1:15,] %>% st_geometry,axes=T, xlim = xlim, ylim = ylim, main = title)} # blank template
    else {plot(pol[1:15,] %>% st_geometry,axes=T, xlim = xlim, ylim = ylim)}
    plot(pol[16:30,] %>% st_geometry + c(0,1), add = T)
    }
# base vertical
base_vert = function(use_title = FALSE, title = '',xlim=c(0,31), ylim=c(0,31), ...) {   #
    if(use_title) {plot('.',axes=T,xlim=xlim,ylim=ylim, main = title)} # blank template
    else {plot('.',axes=T,xlim=xlim, ylim=ylim)}
    for (i in 1:15){
        plot(pol[i,] %>% st_geometry + c(0,i), add = T)
        plot(pol[15+i,] %>% st_geometry + c(0,(15+i)), add = T)
    }}

# more elaborate graphics
pdf=F
if(pdf) pdf('kreft&jetz_graphIII.pdf', width = 8.25, height = 11.75) else dev.new()
par(mfrow = c(2,2),mar = c(2,3,3,2))
# dev.off() # turn on; turn off pdf writer

#### all species blue - red ####
#HORIZ
{plot(c(),axes=T,xlim=c(0,31), ylim=c(-6,6))#, main = 'S1:S15, N16:N30') # blank template
plot(pol[15:1,] %>% st_geometry, col = rgb(0,0,1,.3),add = T)
plot((pol[16:30,] %>% st_geometry) - c(0,0) , col = rgb(1,0,0,.2), add = T)}
#VERT
{plot('.',axes=T,xlim=c(0,31),ylim=c(0,30))#, main = 'S1:S15, N16:N30')# blank template
for (i in 0:14){
    plot(pol[i+1,] %>% st_geometry + c(0,i), add = T, col= rgb(0,0,1,.5))
    plot(pol[i+16,] %>% st_geometry + c(0,15+i), add = T, col = rgb(1,0,0,.5))}
}
#### Color plot templates (functions) ####
# dev.new(); par(mfrow=c(3,2))
#### Biotic elements - color plots ####
# Max_level = 3 #
# par(mfrow = c(4,2), mar = c(2,3,3,2))
interval = 0
base_horiz(use_title = F, title = 'max_depth = 3') # use_title = F to main title
{   for(i in 15:11) plot(pol[i,] %>% st_geometry+interval, col = rgb(0,1,0,.5), add = T)  # green
    for(i in 10:6) plot(pol[i,] %>% st_geometry+interval, col = rgb(0,0,1,.5), add = T)  # blue
    for(i in 16:20) plot(pol[i,] %>% st_geometry+interval + c(0,+1) , col = c(rgb(1,1,0,.5)), add = T)  # yellow
    for(i in 21:25) plot(pol[i,] %>% st_geometry+interval + c(0,+1) , col = c(rgb(1,0,0,.5)), add = T)  # red
    for(i in 28:30) plot(pol[i,] %>% st_geometry+interval + c(0,+1) , col = c(rgb(1,0,1,.5)), add = T) # pink
}  
base_vert(use_title = F, title = 'max_depth = 3')#, ylim = c(0,40))
{   for(i in 6:10) plot(pol[i,] %>% st_geometry+interval + c(0,i), add = T, col = rgb(0,0,1,.7))
    for(i in 11:15) plot(pol[i,] %>% st_geometry+interval +c(0,i), add = T, col = rgb(0,1,0,.7))
    for(i in 16:20) plot(pol[i,] %>% st_geometry+interval +c(0,i) , col = c(rgb(1,1,0,.7)), add = T)
    for(i in 21:25) plot(pol[i,] %>% st_geometry+interval +c(0,i) , col = c(rgb(1,0,0,.7)), add = T)
    for(i in 28:30) plot(pol[i,] %>% st_geometry+interval +c(0,i) , col = c(rgb(1,0,1,.7)), add = T)
}
base_horiz(use_title = F, title = 'max_depth = 5')
{   for(i in 15:1) plot(pol[i,] %>% st_geometry+interval + c(0,-1) , col = rgb(.5,.5,1,.5), add = T)
    # for(i in 10:1) plot(pol[i,] %>% st_geometry+interval + c(0,-1) , col = c(rgb(.3,0,.8,.7)), add = T)
    for(i in 15:11) plot(pol[i,] %>% st_geometry+interval , add = T, col = rgb(0,1,0,.5))
    for(i in 10:6) plot(pol[i,] %>% st_geometry+interval, add = T, col = rgb(0,0,1,.4))
    for(i in 16:20) plot(pol[i,] %>% st_geometry+interval + c(0,+1) , col = c(rgb(1,1,0,.5)), add = T)
    for(i in 21:26) plot(pol[i,] %>% st_geometry+interval + c(0,+1) , col = c(rgb(1,0,0,.5)), add = T)
    for(i in 16:30) plot(pol[i,] %>% st_geometry+interval + c(0,+2) , col = c(rgb(.5,.5,0,.4)), add = T)
}
base_vert(use_title = F,title = 'max_depth = 5', ylim= c(0,47))
{   for(i in 1:15) plot(pol[i,] %>% st_geometry+interval + c(0, 16+i), add = T, col = rgb(.5,.5,1,.7))
    for(i in 6:10) plot(pol[i,] %>% st_geometry+interval +c(0,i), add = T, col = rgb(0,0,1,.7))
    for(i in 11:15) plot(pol[i,] %>% st_geometry+interval +c(0,i), add = T, col = rgb(0,1,0,.7))
    for(i in 16:20) plot(pol[i,] %>% st_geometry+interval +c(0,i) , col = c(rgb(1,1,0,.7)), add = T)
    for(i in 21:26) plot(pol[i,] %>% st_geometry+interval +c(0,i) , col = c(rgb(1,0,0,.7)), add = T)
    for(i in 16:30) plot(pol[i,] %>% st_geometry+interval + c(0,16+i) , col = c(rgb(.5,.5,0,.7)), add = T)
    # for(i in 28:30) plot(pol[i,] %>% st_geometry+interval +c(0,i) , col = c(rgb(1,0,1,.7)), add = T)
}
base_horiz(use_title = F,title = 'max_depth = 7')
{   for(i in 15:1) plot(pol[i,] %>% st_geometry+interval + c(0,-2) , col = rgb(.5,.5,1,.5), add = T)
    for(i in 15:5) plot(pol[i,] %>% st_geometry+interval + c(0,-1) , add = T, col = rgb(.3,0,.8,.5))
    for(i in 15:11) plot(pol[i,] %>% st_geometry+interval , add = T, col = rgb(0,1,0,.5))
    for(i in 10:5) plot(pol[i,] %>% st_geometry+interval, add = T, col = rgb(0,0,1,.5))
    for(i in 16:20) plot(pol[i,] %>% st_geometry+interval + c(0,1) , col = rgb(1,1,0,.5), add = T)
    for(i in 21:26) plot(pol[i,] %>% st_geometry+interval + c(0,1) , col = c(rgb(1,0,0,.5)), add = T)
    for(i in 16:30) plot(pol[i,] %>% st_geometry+interval + c(0,3) , col = c(rgb(.5,.5,0,.5)), add = T)
    for(i in 16:26) plot(pol[i,] %>% st_geometry+interval + c(0,2) , col = c(rgb(1,.5,0,.5)), add = T)
}
base_vert(use_title = F,title = 'max_depth = 7', ylim= c(0,47))
{   for(i in 1:15) plot(pol[i,] %>% st_geometry+interval + c(0,16+i) , col = c(rgb(.5,.5,1,.5)), add = T)
    for(i in 5:15) plot(pol[i,] %>% st_geometry+interval + c(0,16+i), col = rgb(.3,0,.8,.5),add=T)
    # for(i in 1:10) plot(pol[i,] %>% st_geometry+interval + c(0,15 + i), add = T, col = rgb(.3,0,.8,.7))
    for(i in 5:10) plot(pol[i,] %>% st_geometry+interval +c(0,i), add = T, col = rgb(0,0,1,.7))
    # for(i in 1:15) plot(pol[i,] %>% st_geometry+interval + c(0,15+i), add = T, col = rgb(.5,.5,1,.7))
    for(i in 11:15) plot(pol[i,] %>% st_geometry+interval +c(0,i), add = T, col = rgb(0,1,0,.7))
    for(i in 16:20) plot(pol[i,] %>% st_geometry+interval +c(0,i) , col = c(rgb(1,1,0,.7)), add = T)
    for(i in 21:26) plot(pol[i,] %>% st_geometry+interval +c(0,i) , col = c(rgb(1,0,0,.7)), add = T)
    for(i in 16:30) plot(pol[i,] %>% st_geometry+interval + c(0,16+i) , col = c(rgb(.5,.5,0,.7)), add = T)
    for(i in 16:26) plot(pol[i,] %>% st_geometry+interval + c(0,16+i) , col = c(rgb(1,.5,0,.7)), add = T)
}
base_horiz(use_title = F,title = 'max_depth = 10')
{   for(i in 15:1) plot(pol[i,] %>% st_geometry+interval , col = rgb(.5,.5,1,.5), add = T)
    for(i in 16:30) plot(pol[i,] %>% st_geometry+interval + c(0,1), col = c(rgb(.5,.5,0,.5)), add = T)}
base_vert(use_title = F,title = 'max_depth = 10')
{   for(i in 1:15) plot(pol[i,] %>% st_geometry+interval + c(0,i) , col = c(rgb(.5,.5,1,.5)), add = T)
    for(i in 16:30) plot(pol[i,] %>% st_geometry+interval + c(0,i) , col = c(rgb(.5,.5,0,.7)), add = T)}

if(pddf) dev.off() # turn on; turn off pdf writer

#### SUMMARIZING RESULTS ####
biotic_summary %>% group_by(max_depth_set) %>% summarise(n())
