# Supplementary material for: Spatial Congruence Analysis (SCAN): A method for detecting biogeographical patterns based on species range congruences
Source: PLoS One. 2021 May 20;16(5):e0245818. doi: 10.1371/journal.pone.0245818 (PMC8136640; doi:10.1371/journal.pone.0245818)
Supplement: Attachment — Submitted filename: Response to Reviewers.docx [file pone.0245818.s011.docx]

Manaus, 02 May 2021

Dear Academic Editor Bruno Bellisario:

We are very pleased with the results of the revision process - the paper was greatly improved by the suggestions and comments provided by the reviewers. In this last round, we tried to follow accurately the suggestions of the first reviewer. In addition, we made some small modifications of our own to improve clarity in a few specific sentences, with no major content modifications. The points raised by the reviewer are listed below and commented regarding their new configuration in the updated manuscript. All changes can be tracked in its commented version.

This whole revision and correction process led to a significant improvement in the manuscript. For this, we are especially grateful to both reviewers. Although we noted that they chose to remain anonymous, we are very eager to thank them publicly. We wonder if you might ask them to reconsider revealing their names so we might acknowledge their contributions specifically in the acknowledgments. After these modifications, the manuscript is ready for acceptance. Please let us if there is any pending problem. By the rules of our graduate program, CG requires formal acceptance of the manuscript to schedule his Ph. D. defense.

Thank you again for your valuable insights and kind conduction of the whole process.

With warm regards,

Cassiano Gatto & Mario Cohn-Haft

Comments and responses:

Reviewer #1: I congratulate the authors for their excellent revision of the manuscript. I have greatly appreciated their effort to integrate all comments by both referees. As regards my own, the authors have included virtually all my suggestions and corrections. So, I do not have further major comments.

Minor comments

- I suggest to not use the word “community” (which is typically used at smaller scales in ecological contexts), but the more appropriate words “floras” and “faunas” (which refer to larger species assemblages). For example: line 25 “in a community” -> “in a flora or fauna”; line 445: communities -> Floras and faunas

Response: lines 23, 170, 274, 295, 297, 322, 366, 417, 427, 429, 443, 651, 671, 672 were modified. The 'community' term was deleted or replaced by other context-dependent terms in each case.

- Line 75: The “use of indexes”. Do you mean similarity coefficients? In such case, they are part of clustering techniques. I think that the sentence might be simpler, clearer and more comprehensive as follows: “The use of clustering and grid-based bipartite network approaches etc.”

Response: we followed the suggestion

- 77: information-bearing unit -> information-bearing units

Response: fixed

- 79: “may be biased by the use of standard cluster-based methods” -> “may produce contrasting results according to specific clustering method used”

Response: we followed the suggestion

- 87: allow -> allows

Response: Fixed

- 92-93: Delete “Areas identified can be seen as analogous to chorotypes”. It is unnecessary and creates confusion.

Response: Deleted

- 102-103: Delete “that have overlapping distributions”. It is unnecessary and creates confusion.

Response: Deleted

- 108. Delete “We show that SCAN can solve specific biogeographical problem cases as other methods recently proposed”. It is unclear and not useful

Response: Deleted

- 109-112. I suggest rephrasing as follows: “SCAN’s mathematically simple and intuitive approach of direct range map comparisons offers an alternative to other available methods, using different computational or conceptual bases, and provides new parameters and metrics that can be useful in interpreting biogeographical patterns”.

Response: Rephrased as suggested

- 202: Please, explain which these default setting values are (only for CT a value is given, 0.1). Also, try to provide some justification (theoretical or empirical) for the proposed default setting values.

Response: We expanded this paragraph to accommodate a more detailed presentation of these parameters, and their default values, and a proper justification (see 195 Control parameters).

- 228: “constitutes the chorotype” -> constitutes a chorotype

Response: Fixed

- 325. bird community -> avifauna

Response: rephrased

- 449 delete space after [58]; 450 delete space after [37]

Resp: Deleted

- For consistency, change “biogeographic” to “biogeographical” and “chorologic” to “chorological” in all instances

Resp: Replaced in all instances

- Although the author removed any reference to “biotic element”, this expression is still present at places, and the word “element” is used in an unclear way: see lines 296, 300-301, 416, 475, 665, 666, 669, 717

Response: Occurrences of the term element were replaced by equivalent terms, such as pattern, or chorotypes, according to the diverse contexts in which it appeared.

- Finally, appreciate the way fuzzy approaches are discussed, as they are uncommonly used and cited. I wonder if the use of medoids to identify representative species (Fattorini, S. 2007, A statistical method for idiographic analyses in biogeographical research. Diversity and Distributions, 13: 836-844. https://doi.org/10.1111/j.1472-4642.2007.00400.x) might represent a fuzzy approach connecting the “chorological” and “regionalization” programmes.

Response: Both analyses compare the distances intra and inter cluster. While the medoid approach chooses the most ‘representative’ species, the fuzzy approach calculates how much these clusters have species with multiple potential membership. But their background ideas are similar, comparisons between clusters. The identification of representative species in SCAN follows another path. It only chooses the species with highest average congruence to the other component species of the chorotype.

Journal Requirements:

Please review your reference list to ensure that it is complete and correct. […]

Response: Following the journal requirements we updated the references fixing some minor problems, such as misspelled author names and extra spaces. 262 – reference number updated; 276 – a more appropriate paper replaced the former reference. The section References was updated and all references manually checked to ensure correct spelling and proper citation of papers.
